# Serum 25-Hydroxyvitamin D and Intact Parathyroid Hormone as Functional Biomarkers of Bone Mass in Early Childhood

**DOI:** 10.1016/j.tjnut.2025.03.022

**Published:** 2025-03-24

**Authors:** Karen M O’Callaghan, Celine Funk, Farzana Fariha, Marium H Nagaria, Alison Dasiewicz, Jennifer Harrington, Abdullah Al Mahmud, Steven A Abrams, Tahmeed Ahmed, Daniel R Moore, Daniel E Roth

**Affiliations:** 1 Department of Nutritional Sciences, King’s College London, London, United Kingdom; 2 Centre for Global Child Health and SickKids Research Institute, Hospital for Sick Children, Toronto, Ontario, Canada; 3 Infectious Disease Division, International Centre for Diarrhoeal Disease Research, Bangladesh, Dhaka, Bangladesh; 4 Department of Pediatrics, Women’s and Children’s Health Network, University of Adelaide, Adelaide, Australia; 5 Nutrition Research Division, International Centre for Diarrhoeal Disease Research, Bangladesh, Dhaka, Bangladesh; 6 Department of Pediatrics, Dell Medical School at the University of Texas at Austin, Austin, TX, United States; 7 Faculty of Kinesiology and Physical Education, University of Toronto, Toronto, Ontario, Canada; 8 Department of Paediatrics, Hospital for Sick Children, University of Toronto, Toronto, Ontario, Canada; 9 Department of Nutritional Sciences, Faculty of Medicine, University of Toronto, Toronto, Ontario, Canada

**Keywords:** 25-hydroxyvitamin D, vitamin D, bone mineral content, bone mineral density, parathyroid hormone, child, low- and middle-income countries, Bangladesh

## Abstract

**Background:**

The 25-hydroxyvitamin D (25(OH)D) concentration at which parathyroid hormone (PTH) concentration plateaus has been considered to benchmark vitamin D deficiency. However, in young children, there is limited evidence for a suppression point in the 25(OH)D–PTH relationship or its relevance to bone mass accrual.

**Objectives:**

To determine whether the threshold of 25(OH)D at PTH suppression in young children is corroborated by associations of 25(OH)D (or PTH) with bone mineral content (BMC) and areal bone mineral density (aBMD).

**Methods:**

In a cross-sectional secondary analysis of data from the BONe and mUScle health in Kids (BONUSKids) study of 4-y-old children in Bangladesh, serum 25(OH)D and intact PTH (iPTH) were analyzed by liquid chromatography–tandem mass spectrometry and a chemiluminescent immunoassay, respectively. BMC and aBMD were measured by dual-energy X-ray absorptiometry. Associations between 25(OH)D, iPTH, and bone outcomes (BMC, BMC z-score, aBMD, and aBMD z-score) were modeled using multivariable-adjusted linear regression and spline models. Model fit was compared using Akaike’s Information Criteria.

**Results:**

Of 534 participants (51% female), 28% had 25(OH)D concentrations <25 nmol/L and 34% had iPTH >6.7 pmol/L. Model fit of the inverse relationship between 25(OH)D and iPTH was optimized with an inflection point at 25 nmol/L [<25 nmol/L: −0.16 pmol/L per 1 nmol/L increase in 25(OH)D; 95% confidence interval (CI): −0.22, −0.10; *P* < 0.001), above which the slope attenuated (≥25 nmol/L: –0.02 pmol/L; 95% CI: −0.04, −0.003; *P* = 0.019]. However, the positive linear associations between 25(OH)D and bone mass outcomes were monotonic (*P* < 0.05), and iPTH was not associated with any bone outcome in adjusted models (*P* > 0.05 for all). Associations were similar in males and females.

**Conclusions:**

Among 4-y-old children in Dhaka, Bangladesh, we did not identify a 25(OH)D threshold to define vitamin D deficiency based on its association with bone mass. However, efforts to raise 25(OH)D to ≥25 nmol/L may be warranted based on the relatively strong inverse association of 25(OH)D with iPTH below this threshold.

This trial was registered at clinicaltrials.gov as #NCT03537443.

## Introduction

The role of vitamin D in calcium absorption and bone mineral metabolism has been well established [1]. Although current thresholds for vitamin D deficiency have been debated [2,3], several expert bodies have proposed a minimum target threshold for circulating 25-hydroxyvitamin D (25(OH)D) between 25 and 50 nmol/L to protect against risk of osteomalacia in adults and rickets in children [[4], [5], [6], [7], [8], [9]]. As such, current dietary reference intakes (DRIs) for vitamin D are based on the dietary vitamin D intake which will maintain circulating 25(OH)D above such thresholds in both children and adults [[5], [6], [7]].

Despite the discovery and quantification of many known vitamin D metabolites in recent decades, 25(OH)D remains the most widely used biomarker to assess vitamin D status [10, 11]. As reviewed by Fleet [12], lower circulating calcium induces the expression of parathyroid hormone (PTH), which upregulates the renal hydroxylation of 25(OH)D to biologically active 1,25-dihydroxyvitamin D (1,25(OH)_2_D) to facilitate the controlled cascade of increased intestinal calcium absorption and renal calcium reabsorption. Insufficient vitamin D stores impair 1,25(OH)_2_D-mediated calcium absorption, leading to secondary hyperparathyroidism and subsequent skeletal calcium resorption [1,12]. Under conditions of optimal calcium balance, PTH is suppressed to physiological levels and excess 1,25(OH)_2_D is converted to inactive 1,24,25(OH)_3_D via autoregulated pathways [13]. This negative feedback loop facilitates the tight regulation of calcium homeostasis, whereby PTH and 25(OH)D are inversely related, and prolonged elevations in 1,25(OH)_2_D and PTH promote the osteoclastic activity that negatively impacts bone mass [12,14].

Although DRIs for vitamin D in infancy and early childhood are based on the prevention of nutritional rickets [4], this clinical condition represents the most severe end of the spectrum of vitamin D deficiency [8]. An inflection point in the nonlinear 25(OH)D–PTH relationship, referred to as a PTH suppression point, may identify a 25(OH)D threshold below which subclinical vitamin D deficiency (lower 25(OH)D) most potently contributes to suboptimal bone mineralization. At a population level, this 25(OH)D threshold may, therefore, be used as a risk limit to identify a subgroup of children who are most likely to benefit from interventions to improve vitamin D status. However, there is inconsistent evidence regarding the 25(OH)D threshold corresponding to PTH suppression in this age group; previous research has suggested a 25(OH)D threshold ranging between 20 and 60 nmol/L [[15], [16], [17]], whereas others have observed monotonic linear relationships between these biomarkers [18]. As previously summarized [15], between-study comparisons are limited by heterogeneity in the population distributions of 25(OH)D and the attenuation of the inverse relationship between PTH and 25(OH)D in populations with relatively high 25(OH)D concentrations. Additionally, as the changes in circulating calcium that govern PTH secretion are dependent on calcium intake and 1,25(OH)_2_D-mediated calcium absorption, the threshold of 25(OH)D below which PTH more rapidly rises may be dependent on habitual calcium intake, which varies across populations.

Even if a vitamin D-dependent PTH suppression point can be established, its importance for bone mass accrual in children and adolescents is uncertain [18], including whether or not the 25(OH)D (or PTH) concentration at this inflection point corresponds to a similar inflection point in the relationship between 25(OH)D (or PTH) and bone mass. In a public health context, consistency across these inflection points would support the use of the 25(OH)D and PTH concentrations at their respective inflection points as thresholds to define vitamin D deficiency or elevated PTH, respectively, based on greater risks of inadequate bone mass accrual.

In a cohort of 4-y-old children in Dhaka, Bangladesh, the present study aimed to estimate the cross-sectional associations between 25(OH)D, PTH, and dual-energy X-ray absorptiometry (DEXA)-derived measures of bone mineral content (BMC) and areal bone mineral density (aBMD) to establish a threshold of 25(OH)D, above which PTH is suppressed and the relationship of 25(OH)D with bone mass is substantially attenuated.

## Methods

### Study design and participant eligibility

This cross-sectional analysis leveraged data from the previously completed BONe and mUScle health in Kids (BONUSKids) study in Dhaka, Bangladesh, which was a follow-up study of the Maternal Vitamin D for Infant Growth (MDIG) trial. Detailed methods and primary outcomes for both the BONUSKids study [19] and the MDIG trial [20,21] have been published previously. Briefly, the MDIG trial was a randomized, double-blind, placebo-controlled trial that assessed the effect of prenatal, with or without postpartum, vitamin D_3_ supplementation on the primary outcome of infant linear growth at 1 y of age. Pregnant women enrolled at 17–24 weeks of gestation (*n* = 1300) were randomly assigned to 1 of 5 trial arms including prenatal:postpartum regimens of 0:0, 4200:0, 16,800:0, 28,000:0, or 28,000:28,000 IU vitamin D_3_/wk until 6 mo postpartum [21]. The BONUSKids study included a subset (*n* = 642) of MDIG trial participants to examine the effects of the prenatal:postpartum intervention on DEXA-derived measures of bone mass, body composition, and muscle strength at 4 y of age. Enrolment to the MDIG trial began in 2014, and all data collection for the BONUSKids study was completed by February 2020.

Eligibility criteria for participation in the BONUSKids study included an age range of 45–51 mo and the ability to ambulate without assistance; participants were excluded due to the presence of an orthopedic cast or diagnosis of any developmental disorders that would limit feasible DEXA scanning [19]. Inclusion of a child’s data in the present study was contingent on the availability of a usable DEXA scan and measures of circulating intact PTH (iPTH) and 25(OH)D at 4 y of age.

### Ethics

Ethical approval for the BONUSKids study was granted by the Research Ethics Committees of the Hospital for Sick Children in Toronto (REB# 1000060961) and the International Centre for Diarrhoeal Disease Research in Bangladesh (icddr,b; PR-18041); approval for the present study was obtained following an amendment to the study protocol (REB# 1000060961). Written or thumb-print informed consent was obtained by all participants before commencing the MDIG trial, and additional consent was obtained from caregivers for their child’s participation in BONUSKids study activities at 4 y of age. The BONUSKids study and MDIG trial were registered at clinicaltrials.gov (NCT03537443 and NCT01924013, respectively).

### Data collection

Data related to general health and sociodemographic characteristics were assessed via interviewer-administered questionnaires. Household asset index was used as a proxy of socioeconomic status at the 4-y visit, as determined by claimed ownership of relevant household items and derived using principal component analysis [22]. Procedures for assessing anthropometry, BMC, and aBMD have been described in detail elsewhere [19]. Briefly, height was measured using a stadiometer to the last completed 1 mm (Leicester Height Measure device; Chasmors). Weight was measured using a digital scale to the nearest 50 g (Seca 874; Seca). Anthropometric z-scores of height, weight, and BMI-for-age were generated in accordance with the WHO Child Growth Standards [23]. Full-body DEXA scans were performed on a GE Lunar Prodigy narrow-angle fan-beam DEXA scanner (GE HealthCare) using the enhanced analysis mode on the enCORE software (GE HealthCare and enCORE) as described previously [19]. Total-body-less-head (TBLH; subcranial skeleton from base of neck to feet) measures of BMC and aBMD were used as the outcome measure in line with recommendations from the International Society for Clinical Densitometry [24]. The quality of the DEXA scans was assessed for inclusion as previously described based on the presence of minimal motion artifact [19]. TBLH BMC and aBMD z-scores were calculated using the formulas derived by Crabtree et al. [25] based on data from a multiethnic United Kingdom-based population.

Dietary intake was estimated from a single 24-h dietary recall, administered through a structured interview with the child’s caregiver using the multiple-pass method [26,27]. Given known limitations of single 24-h recalls [[28], [29], [30]], rather than deriving a quantified measure of daily calcium intake, the frequency of dairy product consumption was determined as a proxy for bioavailable calcium intake, whereby each meal, beverage, or snack containing a dairy ingredient was determined as 1 serving, irrespective of serving size. Frequency of dairy intake was extracted from the dietary recall forms by 2 independent reviewers; any disagreements were resolved by consensus.

Nonfasting venous blood samples were maintained on ice until processed to serum and either used for same-day analysis at the Clinical Biochemistry Lab (CBL) at icddr,b or stored at <−70°C until further analysis.

### Laboratory analysis

Serum 25(OH)D was analyzed by liquid chromatography–tandem mass spectrometry (LC–MS/MS) at the Analytical Facility for Bioactive Molecules (Hospital for Sick Children, Toronto, Canada) as described previously [19,21], using National Institute of Standards and Technology (NIST) quality control materials (SRM 972a) and Vitamin D External Quality Assessment Scheme (DEQAS) standards. Chromatographic separation of 25-hydroxyvitamin D_3_ (25(OH)D_3_), 3-epi-25-hydroxyviamin D_3_ (3-epi-25(OH)D_3_), and 25-hydroxyvitamin D_2_ (25(OH)D_2_) was achieved. The lower limit of quantification (LLoQ) for 25(OH)D_3_ and 25(OH)D_2_ was 0.05 and 0.125 nmol/L, respectively; 25(OH)D_2_ concentrations above the LLoQ were not encountered. The LLoQ of 3-epi-25(OH)D_3_ was 0.125 nmol/L; concentrations above the LLoQ were detected in all participants but excluded from analysis due to uncertainty about the biological activity of this epimer [31]. Hence, only 25(OH)D_3_ values are presented. Based on NIST and DEQAS standards, the mean bias and interassay coefficient of variation for 25(OH)D_3_ at 4 y of age was −9.0% and 9.3%, respectively. Vitamin D deficiency was defined as 25(OH)D < 30 nmol/L [5].

Serum iPTH was quantified in fresh (i.e. never frozen) samples at CBL by chemiluminescence immunoassay on an Abbott ARCHITECT i1000SR immunoassay analyzer (Abbott Diagnostics, Ltd) using commercial assay kits (Architect iPTH (100T), kit #08K2529; Abbott Diagnostics, Ltd). The LLoQ for iPTH was 0.42 pmol/L. Elevated PTH was defined as iPTH concentrations > 6.7 pmol/L [32].

### Statistical analysis

This secondary analysis was based on a complete-case analysis of children with all DEXA, 25(OH)D, and iPTH data. Data distributions were visually assessed using histograms and kernel density plots. All outcome variables (i.e. iPTH, and TBLH BMC, aBMD, BMC z-score, and aBMD z-score) had approximately normal distributions, and therefore, transformation was not required. Participant characteristics were summarized collectively for the full cohort and separately by sex. Bivariate relationships between variables were visually assessed using scatterplots with locally weighted regression (LOWESS).

The association between 25(OH)D and iPTH was modeled using unadjusted and multivariable-adjusted piecewise linear regression models, whereby linear spline terms were used to account for expected nonlinearity of the 25(OH)D–iPTH relationship (referred to as the spline model). An inflection point was identified by visual inspection, and a knot was placed at the corresponding 25(OH)D value. In a series of models, the location of the single knot was shifted by 1 nmol/L increments within ±10 nmol/L of the visually identified 25(OH)D–iPTH inflection point [33]. Akaike’s Information Criteria (AIC) was used to determine optimal knot placement for the spline model and for comparison to an unsegmented linear regression model (referred to as “linear model”). The lower and upper bounds of the confidence intervals (CIs) surrounding the assigned target values for NIST 25(OH)D reference material typically fall within a range of 3–5 nmol/L [34]. Therefore, distinguishing among thresholds for 25(OH)D that differ within an accepted error range of ≤5 nmol/L is likely trivial. Hence, if differences in AIC were negligible across models and/or knot placement differed <±5 nmol/L, we favored placement of the knot at a conventional 25(OH)D threshold for vitamin D deficiency (e.g. 25 and 30 nmol/L) [2]. Hypothesized confounders of the 25(OH)D–iPTH relationship were identified using a direct acyclic graph (DAG) approach [35], informed by the published literature and available data (Supplemental Figures 1–3). Primary inferences were based on multivariable models including the following covariates: child sex, BMI-for-age z-score, height-for-age z-score, whole-blood hemoglobin concentration, daily frequency of dairy items consumed, season of blood draw, maternal education attainment, household asset index, and the intervention arm assigned to the child’s mother upon enrolment to the MDIG trial. The slope and inflection point of the exposure–outcome relationship from the adjusted model was visualized using generalized additive models.

Similarly, spline models were used to identify potential inflection points in the relationships between 25(OH)D and each bone outcome (BMC, aBMD, BMC z-score, and aBMD z-score), whereby placement of the knot was initially assessed by visual inspection, and then models were compared using AIC. The observed inflection points in the 25(OH)D–bone outcome relationships were also examined for proximity to the 25(OH)D value at the identified inflection point in the 25(OH)D–iPTH relationship, considering differences ≤±5 nmol/L to be trivial, as described previously. Covariates included in multivariable models were informed by DAGs and were similar to those listed previously; in models with BMC or BMC z-score as the outcome, we further adjusted for bone area, similar to the approach recommended by Prentice et al. [36]. To facilitate interpretation of effect estimates, the relationship between 25(OH)D and bone outcomes was also expressed as an SD change by dividing the effect estimate by the population SD for the outcome variable of interest and multiplying by a factor of 10; effects estimates thereby reflect the SD change in the outcome per 10 nmol/L increase in 25(OH)D.

The relationships between iPTH and each bone outcome were similarly examined by comparison of model fit for corresponding linear and spline models, with iPTH as the independent variable and each bone outcome as a continuous dependent variable; in spline models, knots were placed at 1 pmol/L increments of iPTH and model fit assessed by AIC. Covariates included in multivariable models were similar to those listed previously; however, models that included iPTH as the independent variable were also adjusted for serum 25(OH)D concentrations at 4 y of age. We also tested the statistical interaction between iPTH and 25(OH)D on bone outcomes; however, the interaction term was not statistically significant in unadjusted models (*P* > 0.05 in all models), so it was not included in the final multivariable models.

To visually inspect whether the relationship between 25(OH)D and iPTH was modified by dietary calcium intake, we created a categorical variable to capture the frequency of dairy intake (as a proxy for dietary calcium intake), defined as 0, 1, or >1 dairy item consumed per day and compared the shape of the relationship within each category using scatterplots and LOWESS. To test for effect modification of the 25(OH)D–iPTH relationship by dairy intake, we modeled the statistical interaction between 25(OH)D and frequency of dairy intake (continuous independent variables) on iPTH (continuous dependent variable) as a 2-way interaction within the linear and spline regression models outlined in an earlier section; the interaction term was not statistically significant (*P* > 0.05 in all models) and therefore not included in the final multivariable models. Finally, we stratified by sex to examine consistency of inferences from multivariable-adjusted models in the male and female subgroups, primarily as reflected by an overlap of CIs, whereby spline models for both strata used the same inflection points as identified in the primary analysis.

The following considerations guided our interpretation of the slopes and inflection points in the models described above: *1*) we assumed that a key mechanism by which 25(OH)D affects bone health is via its suppression of PTH synthesis and secretion [1]. *2*) A null or substantially attenuated inverse 25(OH)D–iPTH association beyond an identified inflection point (knot) would signal the point at which iPTH is suppressed, such that further increases in 25(OH)D concentrations above this threshold would not be expected to efficiently yield benefits to bone health; this scenario justifies defining vitamin D deficiency as 25(OH)D concentrations below this threshold. Conversely, an inverse 25(OH)D–iPTH association without an inflection point (i.e. linear slope) would suggest that increases in 25(OH)D may be beneficial but would not provide a basis for defining a relative vitamin D deficiency state. *3*) If inflection points were identified in either (or both) of the 25(OH)D–bone or iPTH–bone relationships, and the 25(OH)D and/or iPTH values at these inflection points were consistent with the values at the 25(OH)D–iPTH inflection point, it would strengthen the evidence in support of using the 25(OH)D (or iPTH) threshold to define vitamin D deficiency (or hyperparathyroidism) as it pertains to bone health. *4*) A positive association between 25(OH)D and bone mass without an inflection point would imply that increases in 25(OH)D may benefit bone health throughout the observed range of 25(OH)D but would not provide a basis to define a vitamin D deficiency threshold or optimal target range for 25(OH)D, particularly given the cross-sectional observational design of the study.

Further supplementary analyses included independent-sample *t* tests to examine whether mean BMC, BMC z-score, aBMD, and aBMD z-score differed for participants with 25(OH)D concentrations above and below the identified 25(OH)D–iPTH inflection point. The analysis was completed using Stata (version 17.1; StataCorp) and R statistical software (version 4.4.1; R Foundation for Statistical Computing).

## Results

Of 642 participants enrolled in the BONUSKids study, 534 (83%) contributed data to the cross-sectional analysis based on availability of a usable DEXA scan and measured 25(OH)D and iPTH concentration (Supplemental Figure 4). There was a similar distribution of males and females and relatively even distribution of participants across the maternal vitamin D intervention arm assigned at enrolment to the MDIG trial (Table 1). Overall, mean 25(OH)D concentrations were low, with 43% and 34% of participants presenting with vitamin D deficiency (25(OH)D < 30 nmol/L) and elevated iPTH (>6.7 pmol/L) at 4 y of age, respectively, and 19% of participants had both vitamin D deficiency and elevated iPTH. Mean anthropometric and BMC and aBMD z-scores were low, as expected based on previous reports in this cohort [19,37]. There was a wide distribution in the frequency of dairy items consumed at 4 y of age, ranging from 0 to 7 items per day (Table 1).

**TABLE 1:** Participant, maternal, and household characteristics, by child sex.

	Full cohort (*n* = 534)	Males (*n* = 261)	Females (*n* = 273)
Participant characteristics			
Age (mo)^1^	49 (47, 51)	49 (47, 51)	49 (47, 51)
Height (cm)^2^	98.6 ± 4.4	99.1 ± 4.3	98.2 ± 4.4
Height-for-age z-score	−1.2 ± 1.0	-1.2 ± 1.0	-1.3 ± 1.0
Weight (kg)	14.3 ± 2.2	14.5 ± 2.2	14.1 ± 2.2
Weight-for-age z-score	−1.2 ± 1.1	-1.2 ± 1.1	-1.2 ± 1.1
BMI-for-age z-score	−0.6 ± 1.0	-0.6 ± 1.0	-0.6 ± 1.0
Total-body-less-head BMC (g)	276 ± 52.1	279 ± 52.8	273 ± 51.3
Total-body-less-head BMC z-score	−0.9 ± 1.0	-1.0 ± 1.0	-0.9 ± 1.0
Total-body-less-head aBMD (g/cm^2^)	0.439 ± 0.042	0.442 ± 0.042	0.436 ± 0.041
Total-body-less-head aBMD z-score	−1.4 ± 1.0	-1.5 ± 1.0	-1.3 ± 1.0
Dairy items consumed per day^3^	1 (0, 7)	1 (0, 7)	1 (0, 5)
Season of blood draw (*n*, %)			
Dec–Feb	180 (34)	89 (34)	91 (33)
Mar–May	106 (20)	49 (19)	57 (21)
Jun–Aug	109 (20)	57 (22)	52 (19)
Sep–Nov	139 (26)	66 (25)	73 (27)
Serum 25(OH)D (nmol/L)	34.7 ± 15.4	35.3 ± 16.2	34.2 ± 14.5
Serum 25(OH)D < 30 nmol/L (*n*, %)	231 (43)	114 (44)	117 (43)
Serum 25(OH)D < 25 nmol/L (*n*, %)	150 (28)	75 (29)	75 (27)
Serum iPTH (pmol/L)	5.9 ± 2.5	5.6 ± 2.4	6.2 ± 2.5
Serum iPTH > 6.7 pmol/L (*n*, %)	181 (34)	75 (29)	106 (39)
Serum 25(OH)D < 30 nmol/L and iPTH > 6.7 pmol/L (*n*, %)	99 (19)	47 (18)	52 (19)
Serum 25(OH)D < 25 nmol/L and iPTH > 6.7 pmol/L (*n*, %)	77 (14)	36 (14)	41 (15)
Maternal and household characteristics			
Maternal height (cm)	150.8 ± 5.5	151.1 ± 5.4	150.6 ± 5.6
Maternal BMI (kg/m^2^)	23.8 ± 4.1	23.8 ± 4.2	23.8 ± 4.0
<25 kg/m^2^ (*n*, %)	351 (66)	169 (65)	182 (67)
≥25 to <30 kg/m^2^ (*n*, %)	140 (26)	69 (26)	71 (26)
≥30 kg/m^2^ (*n*, %)	43 (8.1)	23 (8.8)	20 (7.3)
Maternal level of education^4^^,^^5^			
Secondary incomplete or less	417 (78)	201 (77)	216 (79)
Secondary complete	115 (22)	59 (23)	56 (21)
Asset index quartile^6^^,^^7^			
1 (lowest)	151 (28)	83 (32)	68 (25)
2	68 (13)	30 (12)	38 (14)
3	106 (20)	48 (18)	58 (21)
4	208 (39)	99 (38)	109 (40)
Maternal prenatal:postpartum vitamin D supplementation dose (IU/wk) (*n*, %)			
0:0	102 (19)	44 (17)	58 (21)
2400:0	113 (21)	57 (22)	56 (21)
16,800:0	114 (21)	60 (23)	54 (20)
28,000:0	104 (19)	49 (19)	55 (20)
28,000:28,000	101 (19)	51 (20)	50 (18)

1 Values reported as median (minimum, maximum) (all such values).

2 Values reported as mean ± SD (all such values).

3 Frequency of dairy sources derived from a single 24-h recall.

4 *n* = 532 due to 2 missing values (*n* = 260 for males; *n* = 272 for females).

5 Defined as completion of primary school education, equivalent to ≥10 y of schooling.

6 Determined by ownership of household items, using principal component analysis.

7 *n* = 533 due to 1 missing data (*n* = 260 for males).

**FIGURE 1:**
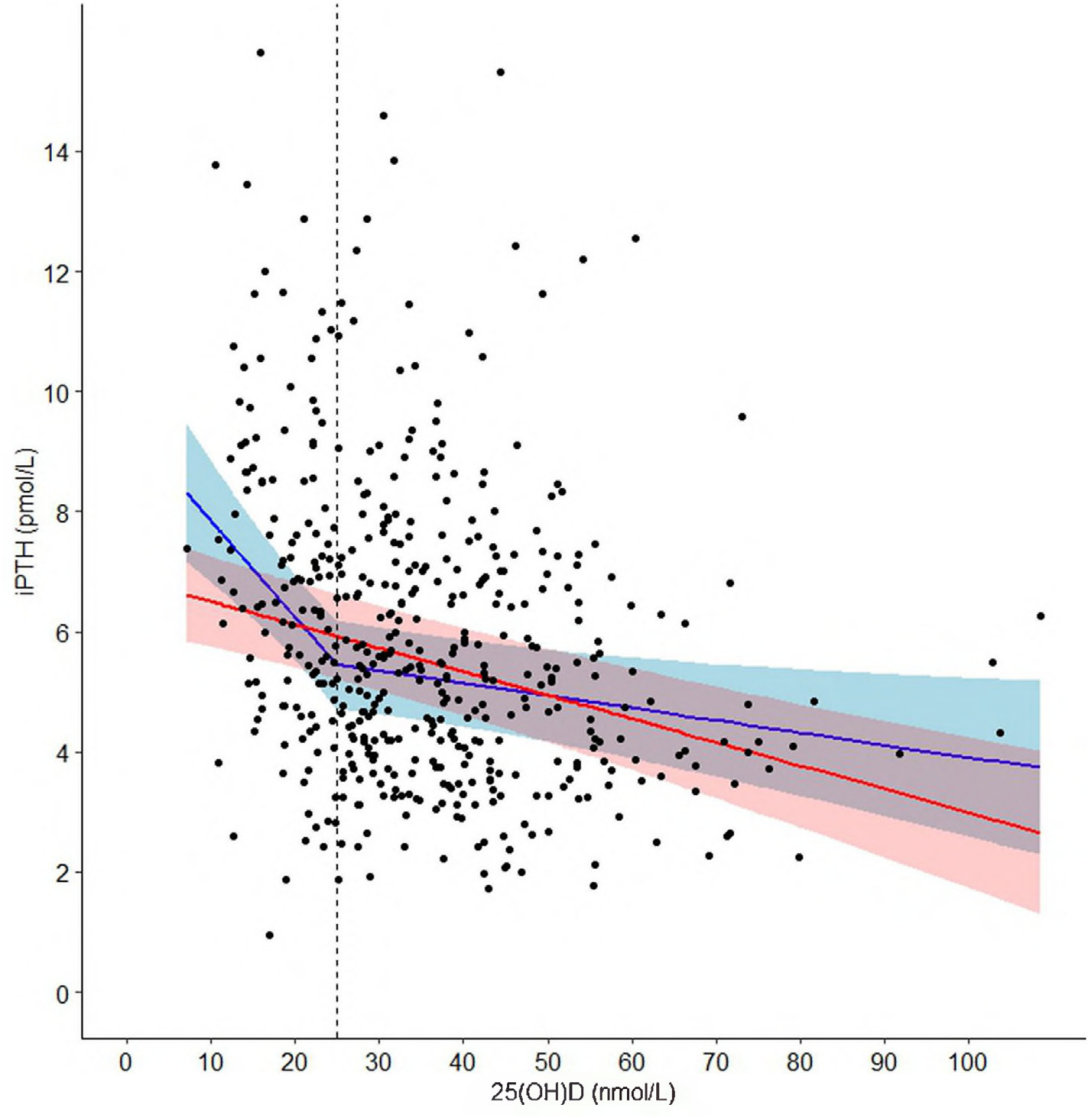
Association between 25(OH)D and iPTH (*n* = 524) based on multivariable (unsegmented) linear regression (red) and spline models (blue). Covariates included the following hypothesized confounders: child sex, BMI-for-age z-score, height-for-age z-score, whole-blood hemoglobin concentration, daily frequency of dairy items consumed, season of blood draw, maternal education attainment, household asset index, and the maternal prenatal and postpartum intervention group assigned at MDIG trial enrolment. For the spline model, the knot was placed at 25 nmol/L of 25(OH)D. 25(OH)D, 25-hydroxyvitamin D; iPTH, intact parathyroid hormone; MDIG, Maternal Vitamin D for Infant Growth.

Nonlinearity of the inverse relationship between 25(OH)D and iPTH was supported by visual inspection, whereby an inflection point in iPTH was identified at 25(OH)D of ∼25 nmol/L (Figure 1). Model fit of the spline model was better than the unsegmented linear model but not improved when spline knots were placed elsewhere within 10 nmol/L, so a knot was retained at 25 nmol/L in the final model (Table 2). The inverse association between 25(OH)D and iPTH was statistically significant on both sides of this inflection point, but the slope of the relationship was substantially attenuated at 25(OH)D concentrations >25 nmol/L, therefore reflecting relatively minor decreases in iPTH as 25(OH)D increases beyond this threshold (Figure 1 and Table 2). Inferences were similar in unadjusted analysis (Supplemental Table 1) and when modeled separately in males and females (Table 3). The shape of the LOWESS curves of the relationship between iPTH and 25(OH)D appeared similar when disaggregated by categories of dairy intake (0, 1, or >1 dairy item consumed per day); however, there was a wide distribution of data around the mean such that the CIs overlapped across all the categories (Supplemental Figure 5). In unadjusted models, the statistical interaction between 25(OH)D and frequency of dairy intake on iPTH concentrations was not significant, either above (*β* for 25(OH)D-by-dairy intake interaction = −0.001 pmol/L; 95% CI: −0.013 to 0.011) or below (*β* for interaction = 0.04 pmol/L; 95% CI: −0.02 to 0.09) the 25 nmol/L inflection point; therefore, multivariable modeling with interactions was not pursued.

**TABLE 2:** Associations of 25-hydroxyvitamin D with intact parathyroid hormone and DEXA-derived bone outcomes^1^.

	Full cohort (*n* = 524)			
	*β*	95% CI	*P*	AIC
iPTH (pmol/L)^2^				
Linear regression	−0.04	−0.05 to −0.02	<0.001	2410
Spline model				
25(OH)D < 25 nmol/L	−0.16	−0.22 to −0.10	<0.001	2397
25(OH)D ≥ 25 nmol/L	−0.02	−0.04 to −0.003	0.019	
TBLH BMC (g)^3^				
Linear regression	0.19	0.08 to 0.29	<0.001	4523
Spline model				
25(OH)D < 25 nmol/L	0.10	−0.39 to 0.58	0.7	4525
25(OH)D ≥ 25 nmol/L	0.20	0.07 to 0.33	0.003	
TBLH BMC z-score^3^				
Linear regression	0.007	0.002 to 0.011	0.007	1265
Spline model				
25(OH)D < 25 nmol/L	−0.004	−0.025 to 0.018	0.7	1266
25(OH)D ≥ 25 nmol/L	0.008	0.002 to 0.014	0.005	
TBLH aBMD (g/cm^2^)^2^				
Linear regression	0.0003	0.0001 to 0.0005	<0.001	−2258
Spline model				
25(OH)D < 25 nmol/L	0.0001	−0.0006 to 0.0009	0.7	−2256
25(OH)D ≥ 25 nmol/L	0.0003	0.0001 to 0.0005	0.001	
TBLH aBMD z-score^2^				
Linear regression	0.008	0.003 to 0.012	<0.001	1115
Spline model				
25(OH)D < 25 nmol/L	0.003	−0.016 to 0.022	0.8	1117
25(OH)D ≥ 25 nmol/L	0.008	0.003 to 0.013	0.001	

25(OH)D, 25-hydroxyvitamin D; aBMD, areal bone mineral density; AIC, Akaike’s Information Criteria; BMC, bone mineral content; DEXA, dual-energy x-ray absorptiometry; iPTH, intact parathyroid hormone; TBLH, total-body-less head.

1 Estimates represent the change in the bone outcome variable per 1 nmol/L increase in 25(OH)D, adjusted for covariates. Lower AIC indicated better model fit. If there were no differences in AIC between the (unsegmented) linear regression and spline model, the linear model was chosen.

2 Covariates in both linear and spline models included the following hypothesized confounders: child sex, BMI-for-age z-score, height-for-age z-score, whole-blood hemoglobin concentration, daily frequency of dairy items consumed, season of blood draw, maternal educational attainment, household asset index, and the maternal prenatal and postpartum intervention group assigned at MDIG trial enrolment.

3 Covariates in both linear and spline models included the following hypothesized confounders: child sex, BMI-for-age z-score, height-for-age z-score, TBLH bone area, whole-blood hemoglobin concentration, daily frequency of dairy items consumed, season of blood draw, maternal educational attainment, household asset index, and the maternal prenatal and postpartum intervention group assigned at MDIG trial enrolment.

**TABLE 3:** Associations of 25-hydroxyvitamin D with intact parathyroid hormone and DEXA-derived bone outcomes, stratified by sex^1^.

	Males (*n* = 255)				Females (*n* = 269)			
	*β*	95% CI	*P*	AIC	*β*	95% CI	*P*	AIC
iPTH (pmol/L)^2^								
Linear regression	−0.04	−0.061 to −0.022	<0.001	1168	−0.03	−0.05 to −0.01	0.005	1260
Spline model				1154				1260
25(OH)D < 25 nmol/L	−0.21	−0.29 to −0.12	<0.001		−0.10	−0.20 to −0.01	0.03	
25(OH)D ≥ 25 nmol/L	−0.02	−0.04 to 0.006	0.16		−0.02	−0.05 to 0.01	0.13	
TBLH BMC (g)^3^								
Linear regression	0.20	0.05 to 0.35	0.009	2216	0.15	−0.01 to 0.32	0.07	2336
Spline model				2218				2338
25(OH)D < 25 nmol/L	−0.02	−0.72 to 0.67	>0.9		0.18	−0.53 to 0.89	0.62	
25(OH)D ≥ 25 nmol/L	0.23	0.05 to 0.41	0.012		0.15	−0.05 to 0.35	0.14	
TBLH BMC z-score^3^								
Linear regression	0.006	−0.0009 to 0.013	0.09	647	0.007	0.0005 to 0.014	0.036	640
Spline model				648				642
25(OH)D < 25 nmol/L	−0.010	−0.042 to 0.021	0.52		−0.002	−0.032 to 0.029	>0.9	
25(OH)D ≥ 25 nmol/L	0.009	0.0002 to 0.017	0.045		0.009	0.0005 to 0.017	0.037	
TBLH aBMD (g/cm^2^)^2^								
Linear regression	0.0003	0.0001 to 0.0006	0.005	−1089	0.0003	0.000003 to 0.0005	0.053	−1142
Spline model				−1087				−1140
25(OH)D < 25 nmol/L	0.00002	−0.001 to 0.001	>0.9		0.0002	−0.0009 to 0.001	0.66	
25(OH)D ≥ 25 nmol/L	0.0004	0.0001 to 0.0007	0.007		0.0003	−0.00005 to 0.0006	0.11	
TBLH aBMD z-score^2^								
Linear regression	0.008	0.003 to 0.014	0.005	550	0.007	0.0002 to 0.013	0.043	592
Spline model				551				595
25(OH)D < 25 nmol/L	0.0004	−0.026 to 0.026	>0.9		0.006	−0.022 to 0.034	0.67	
25(OH)D ≥ 25 nmol/L	0.009	0.003 to 0.016	0.007		0.007	−0.001 to 0.014	0.09	

25(OH)D, 25-hydroxyvitamin D; aBMD, areal bone mineral density; AIC, Akaike’s Information Criteria; BMC, bone mineral content; DEXA, dual-energy x-ray absorptiometry; iPTH, intact parathyroid hormone; TBLH, total-body-less head.

1 Estimates represent the change in the bone outcome variable per 1 nmol/L increase in 25(OH)D, adjusted for covariates. Lower AIC indicated better model fit. If there were no differences in AIC between the (unsegmented) linear regression and spline model, the linear model was chosen.

2 Covariates included in the adjusted models included the following hypothesized confounders: BMI-for-age z-score, height-for-age z-score, whole-blood hemoglobin concentration, daily frequency of dairy items consumed, season of blood draw, maternal education attainment, household asset index, and the maternal prenatal and postpartum intervention group assigned at MDIG trial enrolment.

3 Covariates in both the linear and spline models included the following hypothesized confounders: child sex, BMI-for-age z-score, height-for-age z-score, TBLH bone area, whole-blood hemoglobin concentration, daily frequency of dairy items consumed, season of blood draw, maternal education attainment, household asset index, and the maternal prenatal and postpartum intervention group assigned at MDIG trial enrolment.

**FIGURE 2:**
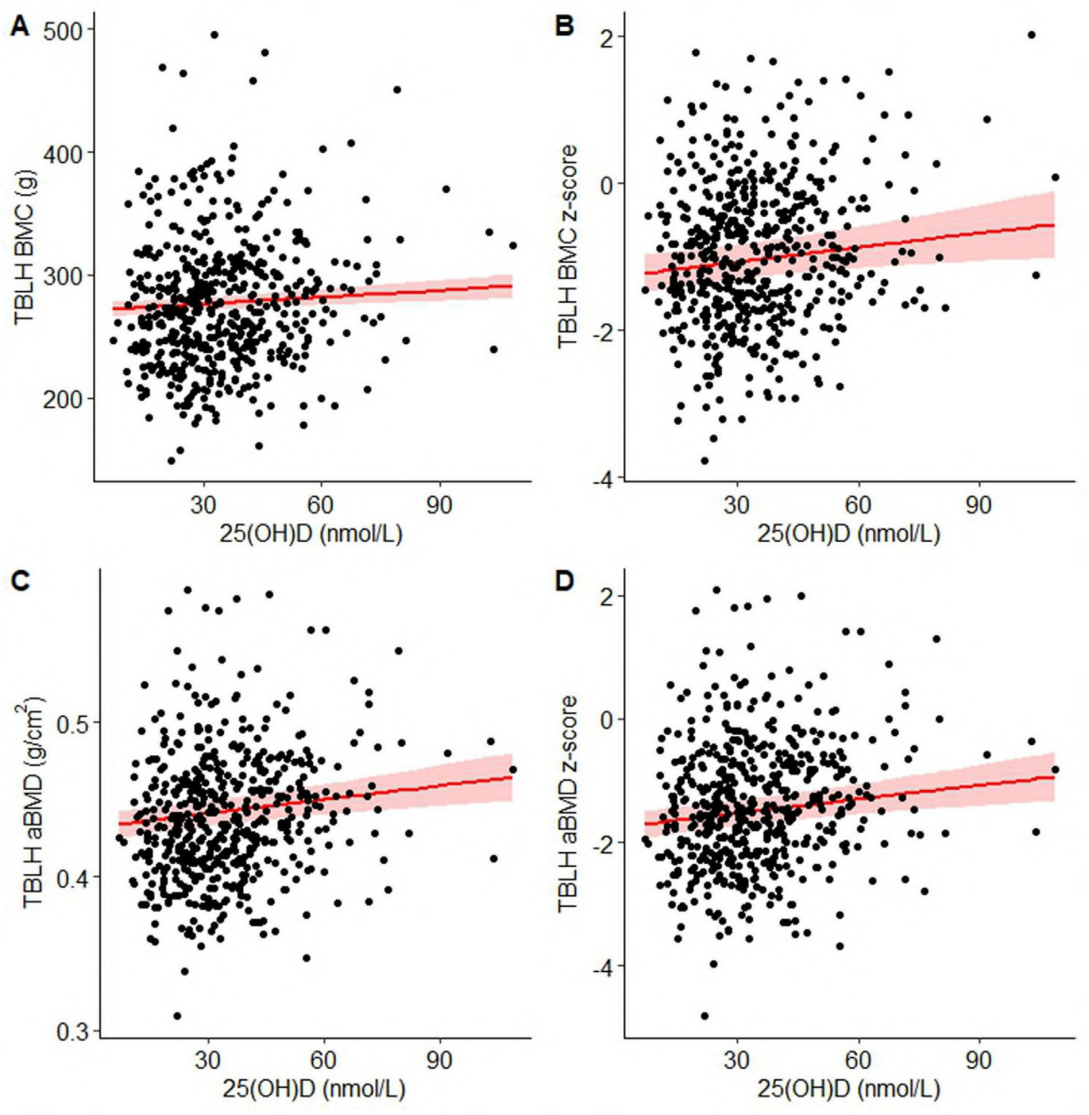
Associations of 25(OH)D with TBLH BMC, BMC z-score, aBMD, and aBMD z-score (*n* = 524), based on multivariable linear regression models. (A) Association between 25(OH)D and BMC. (B) Association between 25(OH)D and BMC z-score. (C) Association between 25(OH)D and aBMD. (D) Association between 25(OH)D and aBMD z-score. Covariates included the following hypothesized confounders: child sex, BMI-for-age z-score, height-for-age z-score, whole-blood hemoglobin concentration, daily frequency of dairy items consumed, season of blood draw, maternal education attainment, household asset index, and the maternal prenatal and postpartum intervention group assigned at MDIG trial enrolment. Models (A) and (B) were additionally adjusted for TBLH bone area. 25(OH)D, 25-hydroxyvitamin D; aBMD, areal bone mineral density; BMC, bone mineral content; MDIG, Maternal Vitamin D for Infant Growth trial; TBLH, total-body-less-head.

On visual inspection, 25(OH)D was positively associated with each of the DEXA outcomes (BMC, BMC z-score, aBMD, and aBMD z-score) without apparent inflection points identified (Figure 2 and Supplemental Figure 6). In unadjusted (Supplemental Table 1) and multivariable linear models (Table 2), there was a positive association between 25(OH)D and BMC, albeit the magnitude of the effect size was small, equating to a 1.9 g, or ∼0.04 SD, increase in BMC per 10 nmol/L increase in 25(OH)D (Table 2). However, based on comparison of model fit, the linear model was preferred for both multivariable-adjusted and -unadjusted analyses (Table 2 and Supplemental Table 1). In multivariable models stratified by child sex, the magnitude of the linear slope for the relationship between 25(OH)D and BMC was similar to the overall estimate in males, whereas in females, it was relatively attenuated (Table 3). Similar to the findings for BMC, there was a weak positive association between 25(OH)D and BMC z-score by linear regression in unadjusted (Supplemental Table 1) and multivariable models (Table 2 and Figure 2) and when stratified by child sex (Table 3). Mean BMC and BMC z-scores were similar for participants with 25(OH)D ≥ and <25 nmol/L (BMC: 279 ± 52 g vs. 270 ± 53 g, respectively; *P* = 0.07; *n* = 534; BMC z-score: −0.90 ± 0.97 vs. −1.03 ± 0.99, respectively; *P* = 0.2; *n* = 534), whereas participants with 25(OH)D ≥25 nmol/L had greater aBMD (0.441 ± 0.041 g/cm^2^ vs. 0.432 ± 0.042 g/cm^2^, respectively; *P* = 0.020; *n* = 534) and greater aBMD z-score (−1.32 ± 1.03 vs. −1.57 ± 1.05, respectively; *P* = 0.015; *n* = 534).

Associations between 25(OH)D and both aBMD and aBMD z-score appeared linear by visual comparison of the regression lines derived from linear and spline models (Figure 2 and Supplemental Figure 6) and AIC values indicating better fit of the unadjusted (Supplemental Table 1) and multivariable linear models (Table 2). In multivariable models, the positive association between 25(OH)D and aBMD was statistically significant (β = 0.003 g/cm^2^ per 10 nmol/L increase in 25(OH)D; 95% CI: 0.001, 0.005; *P* < 0.001), as was the association between 25(OH)D and aBMD z-score (*β* = 0.08 per 10 nmol/L increase in 25(OH)D; 95% CI: 0.03, 0.12; *P* < 0.001), reflecting a <0.1 SD increase in both measures per 10 nmol/L increase in 25(OH)D. Effect estimates were similar in sex-stratified analyses (Table 3).

**FIGURE 3:**
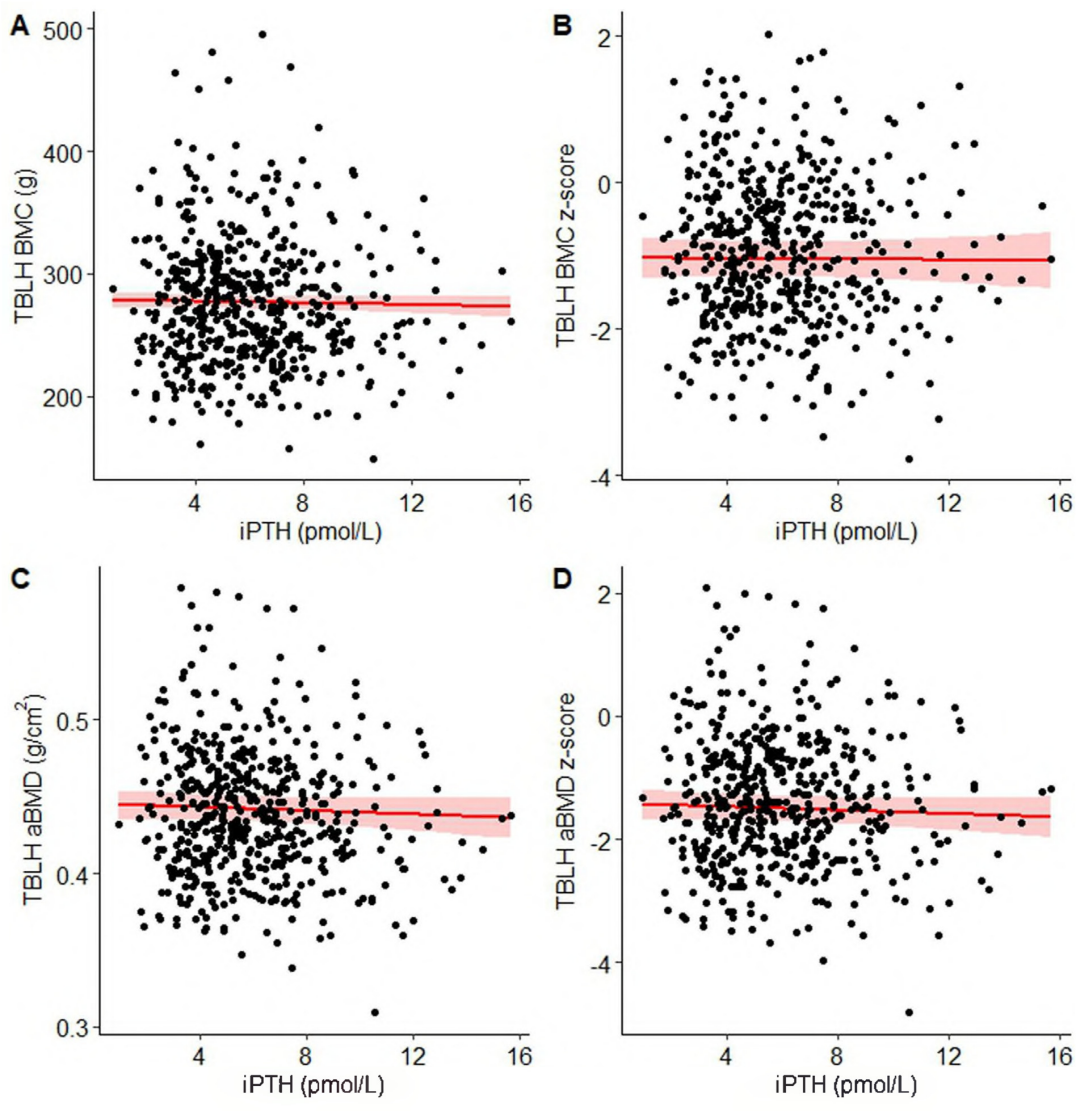
Associations of iPTH with TBLH BMC, BMC z-score, aBMD, and aBMD z-score (*n* = 524), based on multivariable linear regression models. (A) Association between iPTH and BMC. (B) Association between iPTH and BMC z-score. (C) Association between iPTH and aBMD. (D) Association between iPTH and aBMD z-score. Covariates included the following hypothesized confounders: child sex, BMI-for-age z-score, height-for-age z-score, serum 25-hydroxyvitamin D concentration, whole-blood hemoglobin concentration, daily frequency of dairy items consumed, season of blood draw, maternal education attainment, household asset index, and the maternal prenatal and postpartum intervention group assigned at MDIG trial enrolment. Models (A) and (B) were additionally adjusted for TBLH bone area. aBMD, areal bone mineral density; BMC, bone mineral content; iPTH, intact parathyroid hormone; MDIG, Maternal Vitamin D for Infant Growth trial; TBLH, total-body-less-head.

To examine the associations between iPTH and each bone outcome (BMC, BMC z-score, aBMD, and aBMD z-score), spline models were explored with a single knot shifted at 1 pmol/L increments across the range (0.94–15.65 pmol/L) of the iPTH distribution. However, the unsegmented linear model was considered as best fit based on lowest AIC, confirming the absence of inflection points identified by visual inspection (Figure 3 and Supplemental Figure 7). Therefore, only results for the linear model are shown (Table 4). Compared with the unadjusted estimates (Supplemental Table 2), covariate adjustment (including 25(OH)D) attenuated the estimates such that iPTH was not associated with any of the bone outcomes (BMC, BMC z-score, aBMD, and aBMD z-score) in either the full cohort or sex-stratified analyses (Table 4).

**TABLE 4:** Associations of intact parathyroid hormone with DEXA-derived bone outcomes^1^^,^^2^.

	Full cohort (*n* = 534)			Males (*n* = 255)			Females (*n* = 269)		
	*β*	95% CI	*P*	*β*	95% CI	*P*	*Β*	95% CI	*P*
TBLH BMC (g)^3^	−0.34	−1.0 to 0.31	0.30	−0.75	−1.74 to 0.24	0.14	0.002	−0.91 to 0.91	>0.9
TBLH BMC z-score^3^	−0.002	−0.031 to 0.027	0.88	−0.006	−0.05 to 0.04	0.79	0.006	−0.03 to 0.05	0.75
TBLH aBMD (g/cm^2^)	−0.0006	−0.002 to 0.0004	0.26	−0.001	−0.003 to 0.0003	0.11	−0.0003	−0.001 to 0.001	>0.9
TBLH aBMD z-score	−0.014	−0.039 to 0.011	0.28	−0.030	−0.068 to 0.007	0.11	−0.0004	−0.036 to 0.035	>0.9

aBMD, areal bone mineral density; BMC, bone mineral content; DEXA, dual-energy x-ray absorptiometry; iPTH, intact parathyroid hormone; TBLH, total-body-less head.

1 Estimates represent the change in the bone outcome variable per 1 pmol/L increase in iPTH, adjusted for covariates.

2 Covariates included the following hypothesized confounders: child sex (in full cohort only), BMI-for-age z-score, height-for-age z-score, serum 25-hydroxyvitamin D concentration, whole-blood hemoglobin concentration, daily frequency of dairy items consumed, season of blood draw, maternal education attainment, household asset index, and the maternal prenatal and postpartum intervention group assigned at MDIG trial enrolment.

3 Models were additionally adjusted for TBLH bone area.

## Discussion

In this study of 4-y-old children in Dhaka, Bangladesh, we aimed to examine 25(OH)D and iPTH as functional biomarkers of bone health by examining their relationship with one another as well as the strength and shape of their associations with bone mass. We found a nonlinear inverse relationship between 25(OH)D and iPTH with an identifiable inflection point, yet the positive associations between 25(OH)D and DEXA-derived measures of bone mass were monotonic, and iPTH was not associated with any of the bone outcomes. Our findings corroborate ≥25 nmol/L as a relevant target range for 25(OH)D based on suppression of PTH. However, the lack of inflection points in the 25(OH)D–bone outcome relationships, and absence of an iPTH–bone association, limited identification of a meaningful target 25(OH)D (or iPTH) threshold that could be used to guide interventions to optimize bone mass in early childhood in this setting.

Recent pooled analyses from randomized trials did not support the use of PTH to benchmark vitamin D status in children owing to limited evidence for a dose–response relation between vitamin D intake and PTH, and by extension, uncertainties regarding the slope of the 25(OH)D–PTH relationship [10]. However, the inverse association of iPTH with 25(OH)D observed here supports the hypothesis that vitamin D deficiency contributed to elevated iPTH in this cohort. The findings also corroborate the existence of a meaningful inflection point in iPTH at a 25(OH)D concentration of 25 nmol/L, in agreement with a study of neonates in Montreal by Weiler et al. [38]. Nonetheless, there was a lack of a corresponding inflection point in the 25(OH)D–bone associations, and although bone resorption is expected to increase as PTH rises, we did not find an inverse relationship between iPTH and either BMC or aBMD in covariate-adjusted models. Hence, we did not pursue formal mediation analysis to determine the indirect effect of vitamin D status on bone outcomes via attenuation of iPTH. Cross-sectional analyses of the relationship between a single PTH measurement and bone mass may not adequately capture the complex dual osteoanabolic and osteocatabolic functions of PTH; a transient rise in PTH indirectly stimulates bone formation via the Wnt signaling pathway, whereas prolonged elevations promote bone resorption through increased expression of receptor activator of nuclear factor κB ligand (RANKL) relative to osteoprotegerin (OPG) (i.e. RANKL/OPG ratio) [39]. A recent individual participant data meta-analysis suggested that the minimal risk threshold for 25(OH)D for rickets prevention increases at lower calcium intakes [4]. Although our study was not designed to identify risk limits for nutritional rickets, we did not find strong evidence to suggest that a greater dairy intake attenuates the slope of the iPTH–25(OH)D relationship in this population. However, low bone mass in this context may be a corollary of both calcium and vitamin D deficiency [40], and greater accuracy in calcium intake may provide further insights to the interaction between vitamin D and calcium on musculoskeletal health in this population.

Compared with a United Kingdom-based reference population of the same age and sex, we previously reported lower BMC among children in the present cohort [19,37], suggesting an eventual risk of lower peak bone mass. The monotonic linear relationship between 25(OH)D and each of the bone outcomes suggests that bone mass increases as 25(OH)D concentrations increase throughout the range of ∼10–100 nmol/L. Public health interventions (e.g. fortification or supplementation) aimed at increasing 25(OH)D in populations with habitually low vitamin D status may therefore result in commensurate improvements in bone mass and density [41]. However, effect sizes for the association between 25(OH)D and the bone outcomes were small (<0.1 SD per 10 nmol/L increased in 25(OH)D), and the lack of an inflection point hindered identification of a meaningful target 25(OH)D threshold below which the greatest benefits to bone health would be expected. Although vitamin D has an established role in preventing nutritional rickets, there is weak evidence for benefits with respect to other health outcomes [42], and findings from observational studies of the association between 25(OH)D and fracture risk in generally healthy children are inconsistent and subject to confounding [43,44]. Whether a sustained upward shift in the population distribution of 25(OH)D would translate to a greater average peak bone mass and later reduction in fracture risk requires confirmation from trial data with long-term follow-up in pediatric populations.

We acknowledge several limitations of the present analysis. Given the cross-sectional, secondary-use design of this study, we were unable to establish causal relationships between 25(OH)D and bone mass. We used linear spline models and acknowledge that other nonlinear modeling approaches (e.g. polynomials; cubic splines) may have achieved a better model fit; however, a trade-off is that these approaches would not readily yield discrete inflection points. We recognize the potential for residual confounding by general health, lifestyle, or sociodemographic factors, and as such, the magnitude of the effect estimates between biomarkers and the DEXA-derived outcomes should be interpreted with caution. Despite the use of a gold-standard LC–MS/MS method for 25(OH)D analysis, measurement bias exceeded the typically acceptable limit of <5% [45]; however, this systematic error would not be expected to affect the magnitudes of associations. Considering the systematic bias of ∼9% in our study, the relevant threshold for attenuation of iPTH is still expected to be in a range of >20–30 nmol/L. Circulating 25(OH)D is the mostly widely used and accepted biomarker of vitamin D status [11]. Yet, we lacked data on other relevant vitamin D metabolites, including 24,25(OH)_2_D and 1,25(OH)_2_D, which may have given further insight into the vitamin D–bone relationship [46,47]. A low vitamin D metabolite ratio, reflecting diminished 24,25(OH)_2_D and a low ratio of 24,25(OH)_2_D:25(OH)D, is hypothesized to reflect a state of “functional” vitamin D deficiency, such that there is insufficient 25(OH)D availability for catabolism to 24,25(OH)_2_D. This metabolite ratio has therefore been proposed as an alternative to conventional 25(OH)D deficiency cutoffs for identification of individuals who may benefit from vitamin D supplementation, irrespective of their 25(OH)D concentration [47]. Similar to other nutrients (e.g. iron), a panel of biomarkers representing functional vitamin D status may be more relevant for assessing health outcomes, including bone metabolism.

Although efforts toward standardization of PTH measurements are underway [48], like 25(OH)D, differences in assay methodologies for PTH result in wide between-assay variability that limits between-study comparisons, and the intraindividual variation caused by the pulsatile nature of PTH release contributes to preanalytical interindividual variation in PTH [49]. In adults, high calcium intakes have been shown to acutely suppress PTH, whereby PTH remained lower than baseline values ≤5 h post calcium consumption [[50], [51], [52]]. Dietary intake prior to blood sampling may have therefore influenced PTH release in the present study, such that use of fasting samples could have minimized within-child variance in iPTH measurements. Given the lack of an established biomarker that reflects calcium intake and challenges in the assessment of micronutrient intakes in young children [28,29], we estimated dairy consumption as a crude reflection of habitual calcium intake and acknowledge limitations of this approach as compared with quantitative estimates of dietary calcium intake. DEXA is a widely used method for quantifying BMC and aBMD in the pediatric population. However, it produces a 2D estimate of a 3D structure such that aBMD measurements do not fully account for differences in skeletal size; both BMC and aBMD are positively associated with height, thereby necessitating careful interpretation of quantitative measurements of bone mass in populations in which linear growth faltering is common. To facilitate comparison and replication of our findings in other populations, we examined associations of 25(OH)D and PTH with unstandardized measures of BMC and aBMD as well as standardized measures using ethnic- and sex-specific z-score formulas derived by Crabtree et al. [25]. However, methods to account for skeletal size and growth-related differences in BMC and aBMD in this age group lack consensus [53]. Our study population included a subset of participants from a trial cohort, who were willing and available to participate in further follow-up studies. We have previously reported similar sociodemographic characteristics for MDIG trial participants and the subset of participants enrolled in the BONUSKids study [19]; however, we acknowledge the possible effects of attrition bias and reductions in sample size due to missing data at the 4-y visit. Generalizability of our findings to the wider population in Dhaka or elsewhere may therefore be limited. Finally, our findings are not intended to be applied as a 25(OH)D risk limit for nutritional rickets or to reflect a 25(OH)D threshold that would be applicable to children with increased risk of musculoskeletal disorders.

In conclusion, in a population in which childhood undernutrition and low bone mass are common, our findings do not support the presence of a 25(OH)D threshold to define vitamin D deficiency based on its association with low bone mass. However, efforts to raise 25(OH)D to 25 nmol/L or higher may be warranted in young children based on the relatively strong inverse association of 25(OH)D with iPTH below this threshold.

## Author contributions

The authors’ responsibilities were as follows – DER: is the principal investigator and guarantor; DER, KMOC: designed the study; FF: completed data collection; MHN: contributed to data curation; CF, AD, KMOC: performed statistical analysis; AAM: supervised data collection and field study activities in Dhaka; SAA, JH, TA, DRM: provided methodological oversight; KMOC, DER: wrote the manuscript; KMOC: had responsibility for the final content; and all authors: have read and approved the final manuscript.

## Data availability

Data described in the manuscript, code book, and analytic code will be made available upon request to the authors. De-identified individual participant data will be provided for use in secondary data analyses approved by an independent research ethics board, and data requestors may be required to sign a data access agreement.

## Funding

This research was supported by funding from the Canadian Institutes for Health Research (PJT159596) and the Bill and Melinda Gates Foundation (OPP1066764). The funding agencies were not involved in the design, implementation, analysis, or interpretation of the data, or the decision to submit the manuscript for publication.

## Conflict of interest

The authors report no conflicts of interest.
