# Supplementary material for: Serum 25-Hydroxyvitamin D and Intact Parathyroid Hormone as Functional Biomarkers of Bone Mass in Early Childhood
Source: J Nutr. 2025 Mar 24;155(6):1782–94. doi: 10.1016/j.tjnut.2025.03.022 (PMC12264547; doi:10.1016/j.tjnut.2025.03.022)
Supplement: multimedia component 1 [file mmc1.pdf]

Serum 25-hydroxyvitamin D and intact parathyroid hormone as functional biomarkers of bone mass in early childhood

Karen M O'Callaghan, Celine Funk, Farzana Fariha, Marium H Nagaria, Alison Dasiewicz, Jennifer Harrington, Abdullah Al Mahmud, Steven A Abrams, Tahmeed Ahmed, Daniel R Moore and Daniel E Roth.

Online supplementary material

Address correspondence to: Karen M. O'Callaghan, Department of Nutritional Sciences, King's College London, 150 Stamford St, London SE1 9NH, United Kingdom; karen.ocallaghan@kcl.ac.uk

Table of Contents

Supplementary Figure 1: Direct Acyclic Graph depicting hypothesized confounders of the cross-sectional relationship between serum 25(OH)D and PTH at 4 years of age. 1

Supplementary Figure 2: Direct Acyclic Graph depicting hypothesized confounders of the cross-sectional relationship between (A) serum 25(OH)D and bone mineral content and (B) serum 25(OH)D and bone mineral density at 4 years of age. 2

Supplementary Figure 3: Direct Acyclic Graph depicting hypothesized confounders of the cross-sectional relationship between (A) serum PTH and bone mineral content and (B) serum PTH and bone mineral density at 4 years of age. 4

Supplementary Figure 4: CONSORT Flow Diagram of participant enrolment, random assignment and participation in study activities throughout the MDIG trial and follow-up BONUSKids study, by intervention group. 6

Supplementary Figure 5: LOWESS curves for the relationship between 25(OH)D and iPTH by groups of dairy intake. 8

Supplementary Figure 6: Association between 25(OH)D and TBLH BMC, BMC z-score, aBMD and aBMD z-score. 9

Supplementary Figure 7: Association between iPTH and TBLH BMC, BMC z-score, aBMD and aBMD z-score. 11

Supplementary Table 1: Associations of 25(OH)D with iPTH and DXA-derived bone outcomes in unadjusted regression models. 13

Supplementary Table 2: Association between iPTH with DXA-derived bone outcomes in unadjusted regression models. 14

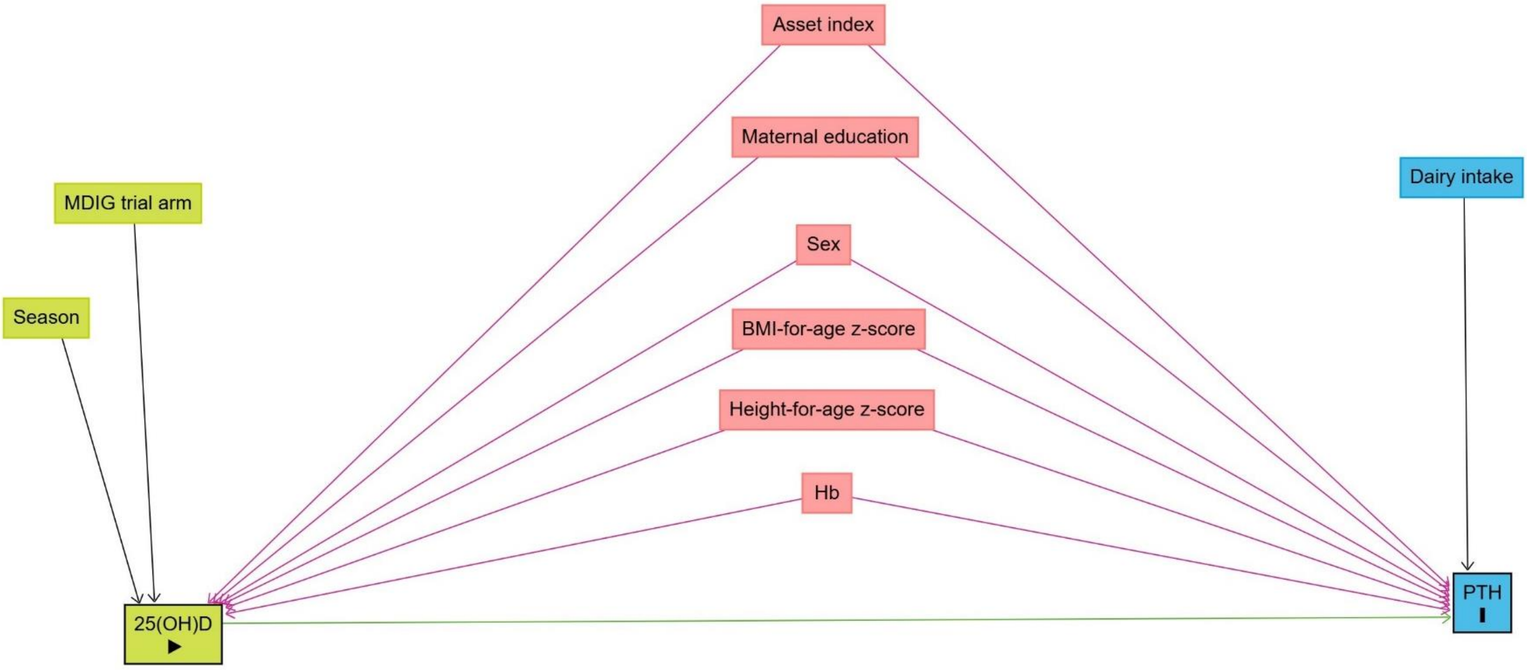

Supplementary Figure 1: Direct Acyclic Graph depicting hypothesized confounders of the cross-sectional relationship between serum 25(OH)D and PTH at 4 years of age.

Figure shows hypothesized relationships between covariates included in multivariable-adjusted analysis of the associations between 25(OH)D as a continuous exposure variable and PTH as a continuous outcome variable. As hypothesized confounders, household asset index and maternal education were considered to represent measures of socioeconomic status; BMI-for-age and height-for-age z-scores represented body size and nutritional status; hemoglobin concentrations were considered as an additional marker of nutritional status. The season of blood sampling and intervention arm assigned to the participant's mother upon enrolment to the MDIG trial were considered as possible determinants of 25(OH)D at 4 years such that these variables were considered to lie on the causal pathway of the exposure-outcome relationship, and hence were included as covariates in adjusted models. Frequency of dairy intake was considered a proxy of bioavailable calcium intake that may have independent effects on PTH, and hence, was included in adjusted models to capture upstream factors. Figure was created using DAGitty (v3.1). 25(OH)D, 25-hydroxyvitamin D; Hb, hemoglobin; PTH, parathyroid hormone.

A

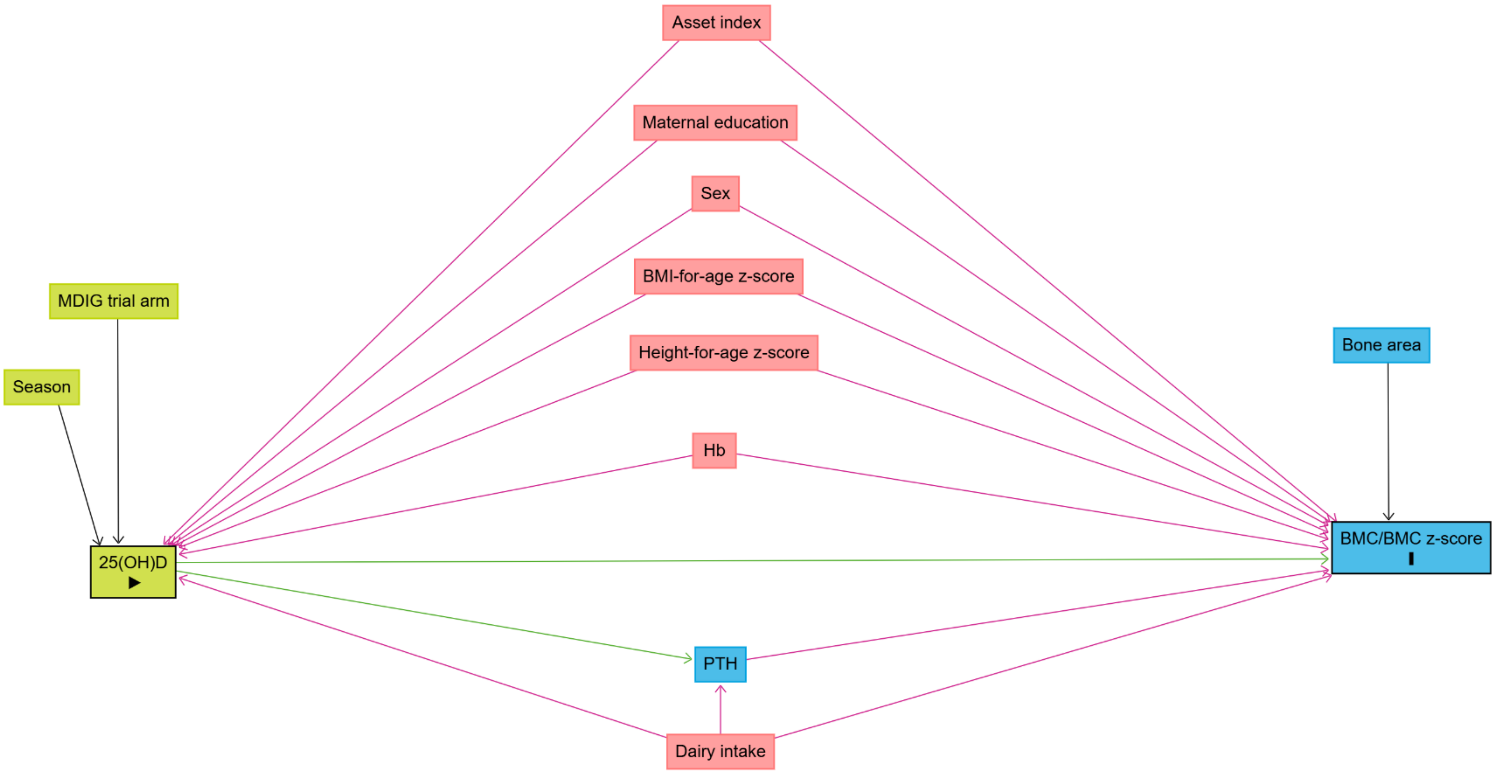
Asset index
Maternal education
Sex
BMI-for-age z-score
Height-for-age z-score
Hb
MDIG trial arm
Season
25(OH)D
Bone area
BMC/BMC z-score
PTH
Dairy intake

B

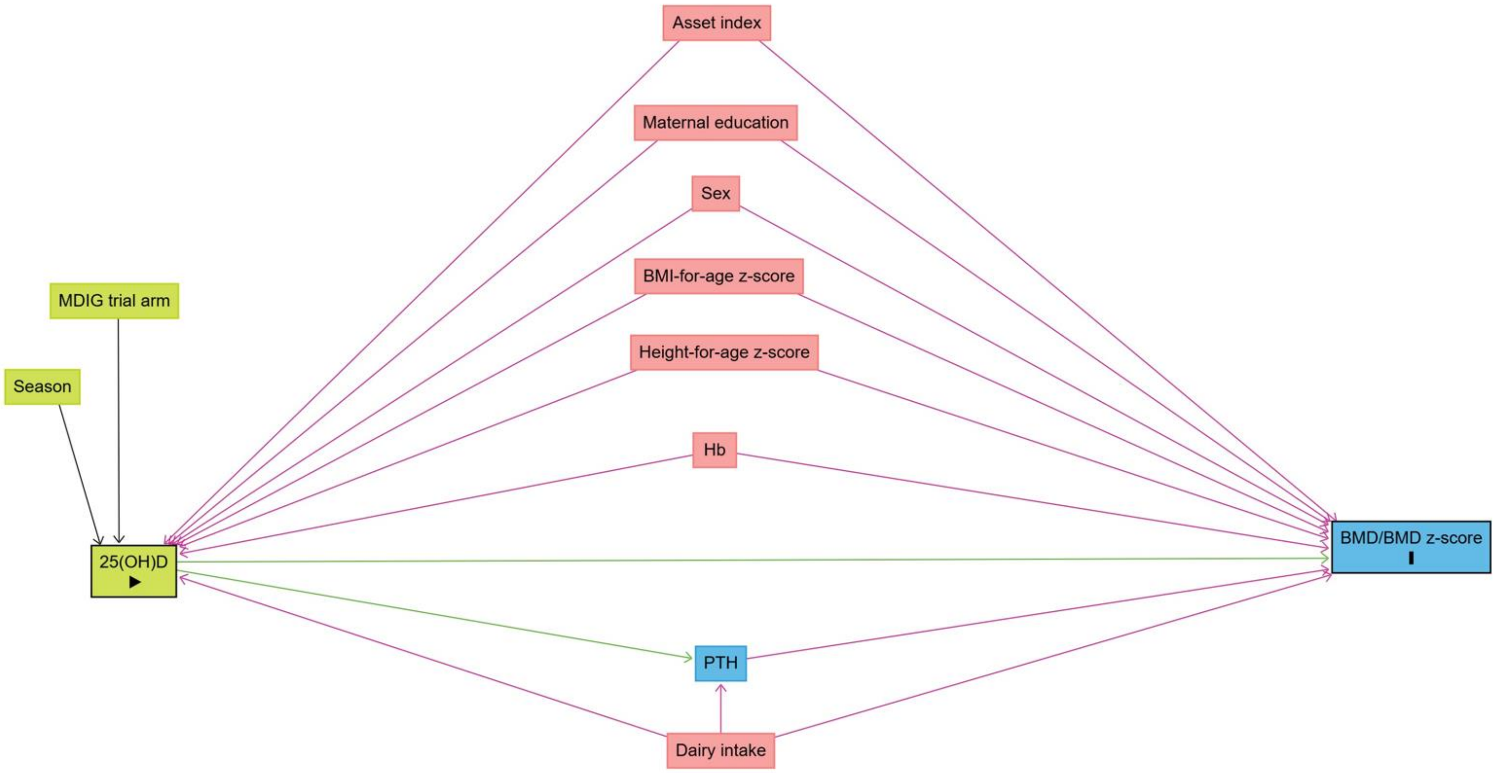
Asset index
Maternal education
Sex
BMI-for-age z-score
Height-for-age z-score
Hb
MDIG trial arm
Season
25(OH)D
BMD/BMD z-score
PTH
Dairy intake

Supplementary Figure 2: Direct Acyclic Graph depicting hypothesized confounders of the cross-sectional relationship between (A) serum 25(OH)D and bone mineral content and (B) serum 25(OH)D and bone mineral density at 4 years of age.

Figure shows hypothesized relationships between covariates included in multivariable-adjusted analysis of the associations between 25(OH)D as a continuous exposure variable and (A) BMC and BMC z-score and (B) BMD and BMD z-score as a continuous outcome variable. As hypothesized confounders, household asset index and maternal education were considered to represent measures of socioeconomic status; BMI-for-age z-score, height-for-age z-score and bone area represented body size and nutritional status; hemogloblin concentrations were considered as an additional marker of nutritional status. The season of blood sampling and intervention arm assigned to the participant's mother upon enrolment to the MDIG trial were considered as possible determinants of 25(OH)D at 4 years such that these variables were considered to lie on the causal pathway of the exposure-outcome relationship, and hence were included as covariates in adjusted models. Frequency of dairy intake was considered a proxy of diet quality that may have either indirect or direct effects on both 25(OH)D and bone mass, and hence, was included in adjusted models. Figure was created using DAGitty (v3.1). 25(OH)D, 25-hydroxyvitamin D; Hb, hemoglobin; PTH, parathyroid hormone.

A

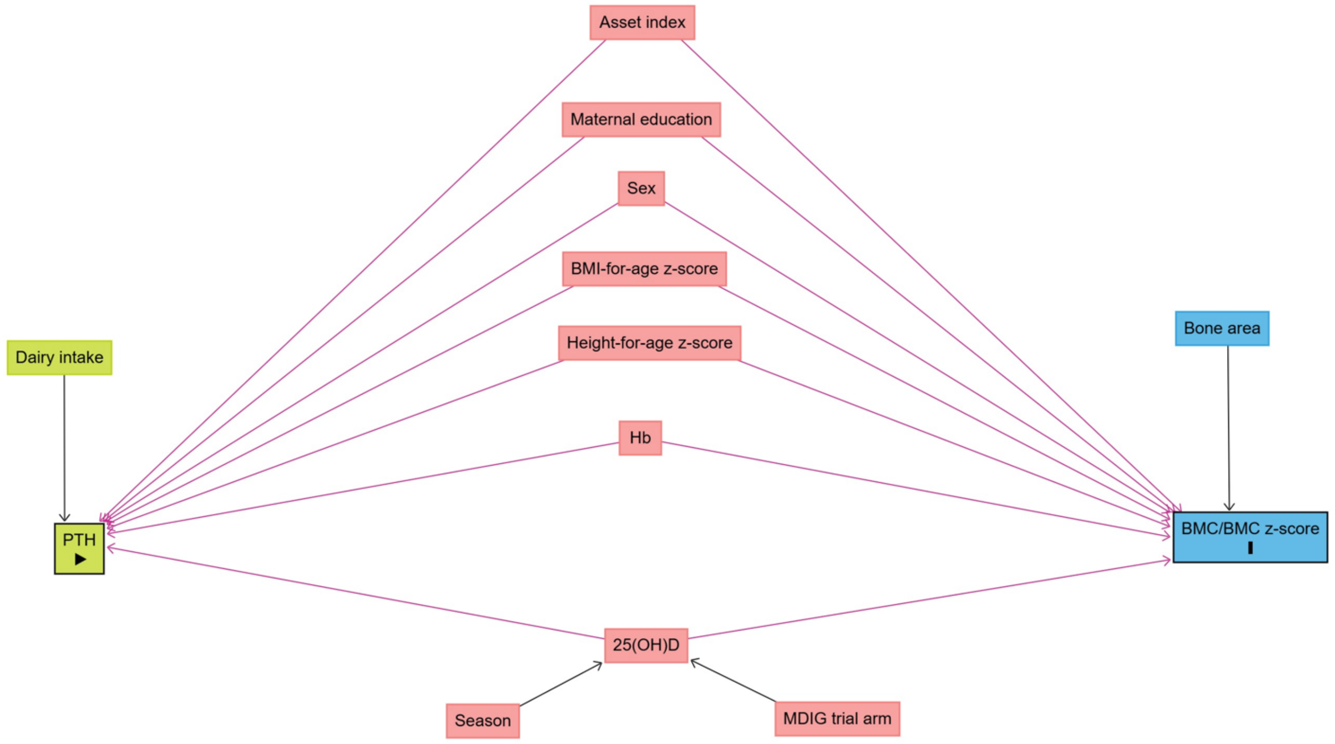

Asset index
Maternal education
Sex
BMI-for-age z-score
Height-for-age z-score
Hb
Dairy intake
PTH
Bone area
BMC/BMC z-score
25(OH)D
Season
MDIG trial arm

B

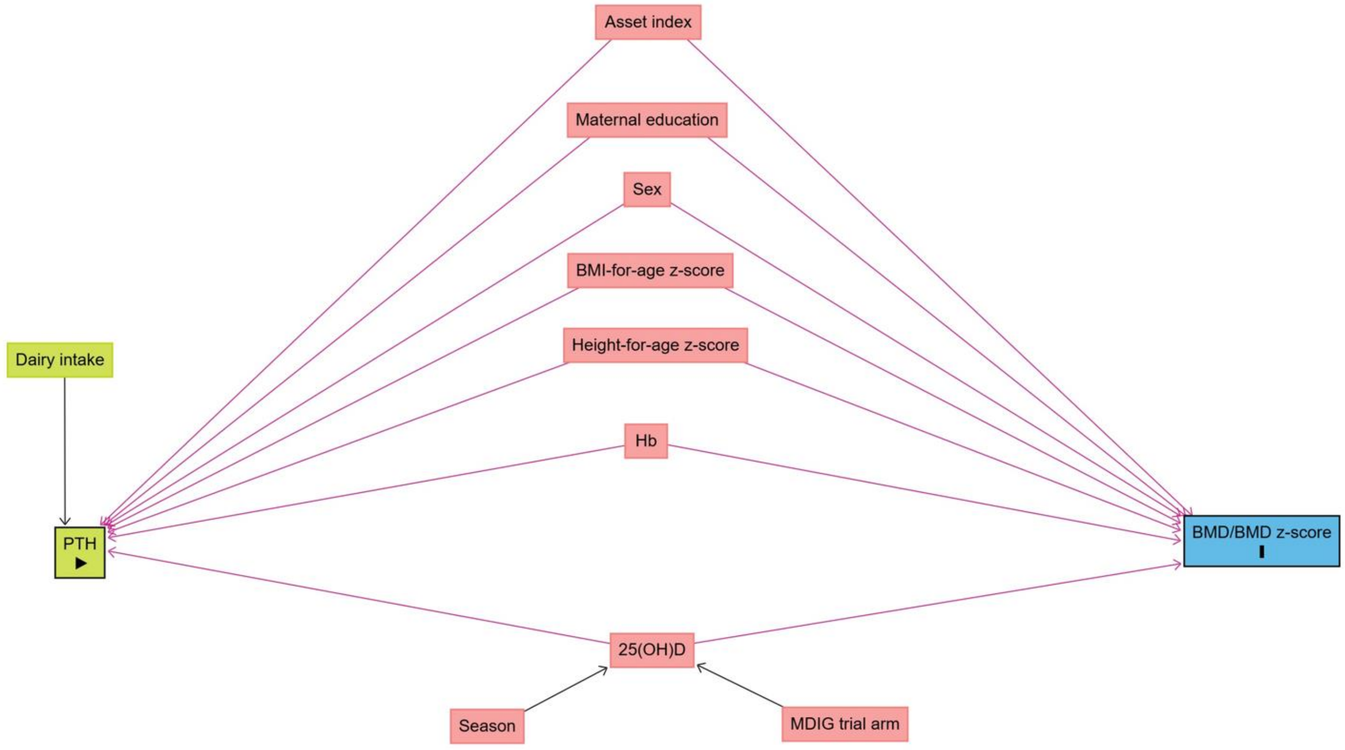

Asset index
Maternal education
Sex
BMI-for-age z-score
Height-for-age z-score
Hb
Dairy intake
PTH
BMD/BMD z-score
25(OH)D
Season
MDIG trial arm

Supplementary Figure 3: Direct Acyclic Graph depicting hypothesized confounders of the cross-sectional relationship between (A) serum PTH and bone mineral content and (B) serum PTH and bone mineral density at 4 years of age.

Figure shows hypothesized relationships between covariates included in multivariable-adjusted analysis of the associations between PTH as a continuous exposure variable and (A) BMC and BMC z-score and (B) BMD and BMD z-score as a continuous outcome variable. As hypothesized confounders, household asset index and maternal education were considered to represent measures of socioeconomic status; BMI-for-age z-score, height-for-age z-score and bone area represented body size and nutritional status; hemogloblin concentrations were considered as an additional marker of nutritional status. 25(OH)D was considered a confounder due to independent effects on PTH and bone outcomes; the season of blood sampling and intervention arm assigned to the participant's mother upon enrolment to the MDIG trial were considered possible determinants of 25(OH)D at 4 years and hence were also included as covariates in adjusted models. Frequency of dairy intake was considered a proxy of bioavailable calcium intake that may have independent effects on PTH, and hence, was included in adjusted models to capture upstream factors. Figure was created using DAGitty (v3.1). 25(OH)D, 25-hydroxyvitamin D; Hb, hemoglobin; PTH, parathyroid hormone.

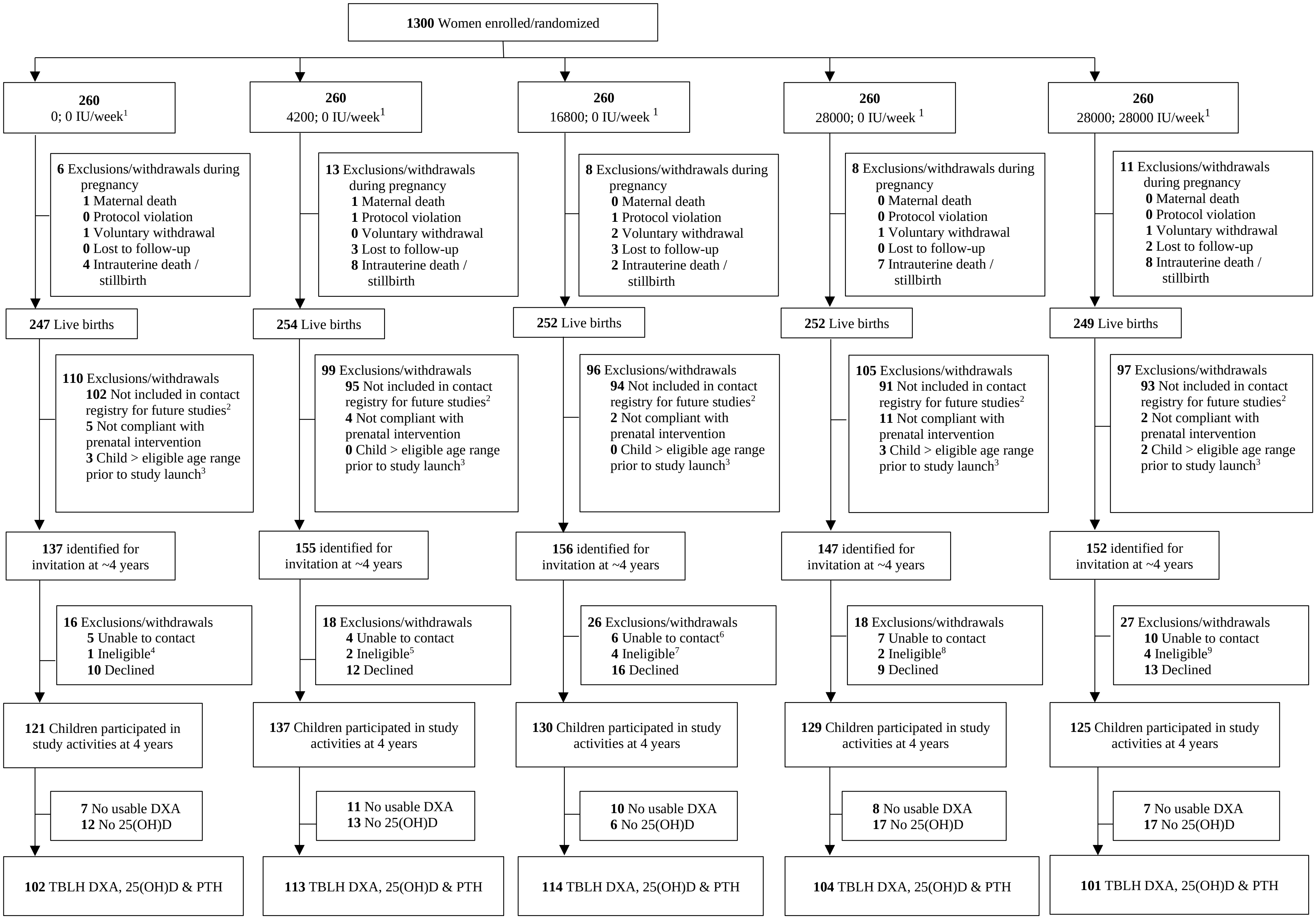
1300 Women enrolled/randomized
260
0; 0 IU/week[1]
6 Exclusions/withdrawals during pregnancy
1 Maternal death
0 Protocol violation
1 Voluntary withdrawal
0 Lost to follow-up
4 Intrauterine death / stillbirth
247 Live births
110 Exclusions/withdrawals
102 Not included in contact registry for future studies[2]
5 Not compliant with prenatal intervention
3 Child > eligible age range prior to study launch[3]
137 identified for invitation at ~4 years
16 Exclusions/withdrawals
5 Unable to contact
1 Ineligible[4]
10 Declined
121 Children participated in study activities at 4 years
7 No usable DXA
12 No 25(OH)D
102 TBLH DXA, 25(OH)D & PTH
260
4200; 0 IU/week[1]
13 Exclusions/withdrawals during pregnancy
1 Maternal death
1 Protocol violation
0 Voluntary withdrawal
3 Lost to follow-up
8 Intrauterine death / stillbirth
254 Live births
99 Exclusions/withdrawals
95 Not included in contact registry for future studies[2]
4 Not compliant with prenatal intervention
0 Child > eligible age range prior to study launch[3]
155 identified for invitation at ~4 years
18 Exclusions/withdrawals
4 Unable to contact
2 Ineligible[5]
12 Declined
137 Children participated in study activities at 4 years
11 No usable DXA
13 No 25(OH)D
113 TBLH DXA, 25(OH)D & PTH
260
16800; 0 IU/week [1]
8 Exclusions/withdrawals during pregnancy
0 Maternal death
1 Protocol violation
2 Voluntary withdrawal
3 Lost to follow-up
2 Intrauterine death / stillbirth
252 Live births
96 Exclusions/withdrawals
94 Not included in contact registry for future studies[2]
2 Not compliant with prenatal intervention
0 Child > eligible age range prior to study launch[3]
156 identified for invitation at ~4 years
26 Exclusions/withdrawals
6 Unable to contact[6]
4 Ineligible[7]
16 Declined
130 Children participated in study activities at 4 years
10 No usable DXA
6 No 25(OH)D
114 TBLH DXA, 25(OH)D & PTH
260
28000; 0 IU/week [1]
8 Exclusions/withdrawals during pregnancy
0 Maternal death
0 Protocol violation
1 Voluntary withdrawal
0 Lost to follow-up
7 Intrauterine death / stillbirth
252 Live births
105 Exclusions/withdrawals
91 Not included in contact registry for future studies[2]
11 Not compliant with prenatal intervention
3 Child > eligible age range prior to study launch[3]
147 identified for invitation at ~4 years
18 Exclusions/withdrawals
7 Unable to contact
2 Ineligible[8]
9 Declined
129 Children participated in study activities at 4 years
8 No usable DXA
17 No 25(OH)D
104 TBLH DXA, 25(OH)D & PTH
260
28000; 28000 IU/week[1]
11 Exclusions/withdrawals during pregnancy
0 Maternal death
0 Protocol violation
1 Voluntary withdrawal
2 Lost to follow-up
8 Intrauterine death / stillbirth
249 Live births
97 Exclusions/withdrawals
93 Not included in contact registry for future studies[2]
2 Not compliant with prenatal intervention
2 Child > eligible age range prior to study launch[3]
152 identified for invitation at ~4 years
27 Exclusions/withdrawals
10 Unable to contact
4 Ineligible[9]
13 Declined
125 Children participated in study activities at 4 years
7 No usable DXA
17 No 25(OH)D
101 TBLH DXA, 25(OH)D & PTH

Supplementary Figure 4: CONSORT Flow Diagram of participant enrolment, random assignment and participation in study activities throughout the MDIG trial and follow-up BONUSKids study, by intervention group. 25(OH)D, 25-hydroxyvitamin D; DXA, dual energy x-ray absorptiometry; TBLH, total-body-less-head; PTH, parathyroid hormone.

[1]Dose received prenatally; postpartum.

[2]Participants who either declined participation, infant died or were lost to follow-up during the postpartum period.

[3]Child was greater than 52 months of age when present study was initiated, and was therefore not contact for participation.

[4]Ineligible due to diagnosis of any developmental disorder that would render difficulty in completion of the DXA scan (*n*=1).

[5]Ineligible as could not be scheduled within the eligible age range (45-51 months of age) (*n*=2).

[6]Includes one child who was screened and eligible but unable to be contacted thereafter.

[7]Ineligible due to inability to bear weight on his/her legs (e.g. wheelchair bound) (*n*=1) or presence of current fracture or break in which his/her limb was supported by an orthopaedic cast (*n*=1) or could not be scheduled within the eligible age range (45-51 months of age) (*n*=2).

[8]Ineligible as could not be scheduled within the eligible age range (45-51 months of age) (*n*=2).

[9]Ineligible due to diagnosis of any developmental disorder that would render difficulty in completion of the DXA scan (*n*=1), unwell at time of scheduled visit (e.g. high temperature) (*n*=1) or could not be scheduled within the eligible age range (45-51 months of age) (*n*=2).

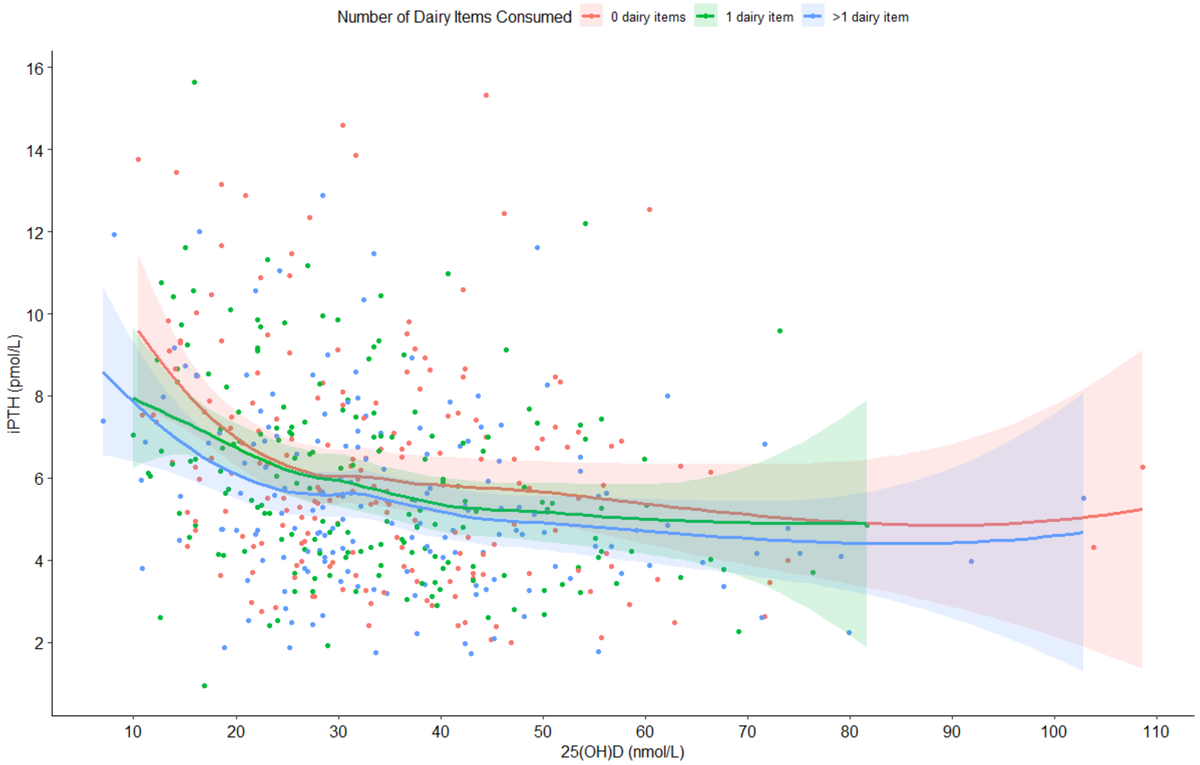

Supplementary Figure 5: LOWESS curves (bandwidth = 0.75) for the relationship between 25(OH)D and iPTH by groups of dairy intake defined as: 0 dairy items consumed per day (n=191), 1 dairy item consumed per day (n=175), and more than 1 dairy item consumed per day (n=168), derived by single 24-hour recall. 25(OH)D, 25-hydroxyvitamin D; iPTH, intact parathyroid hormone.

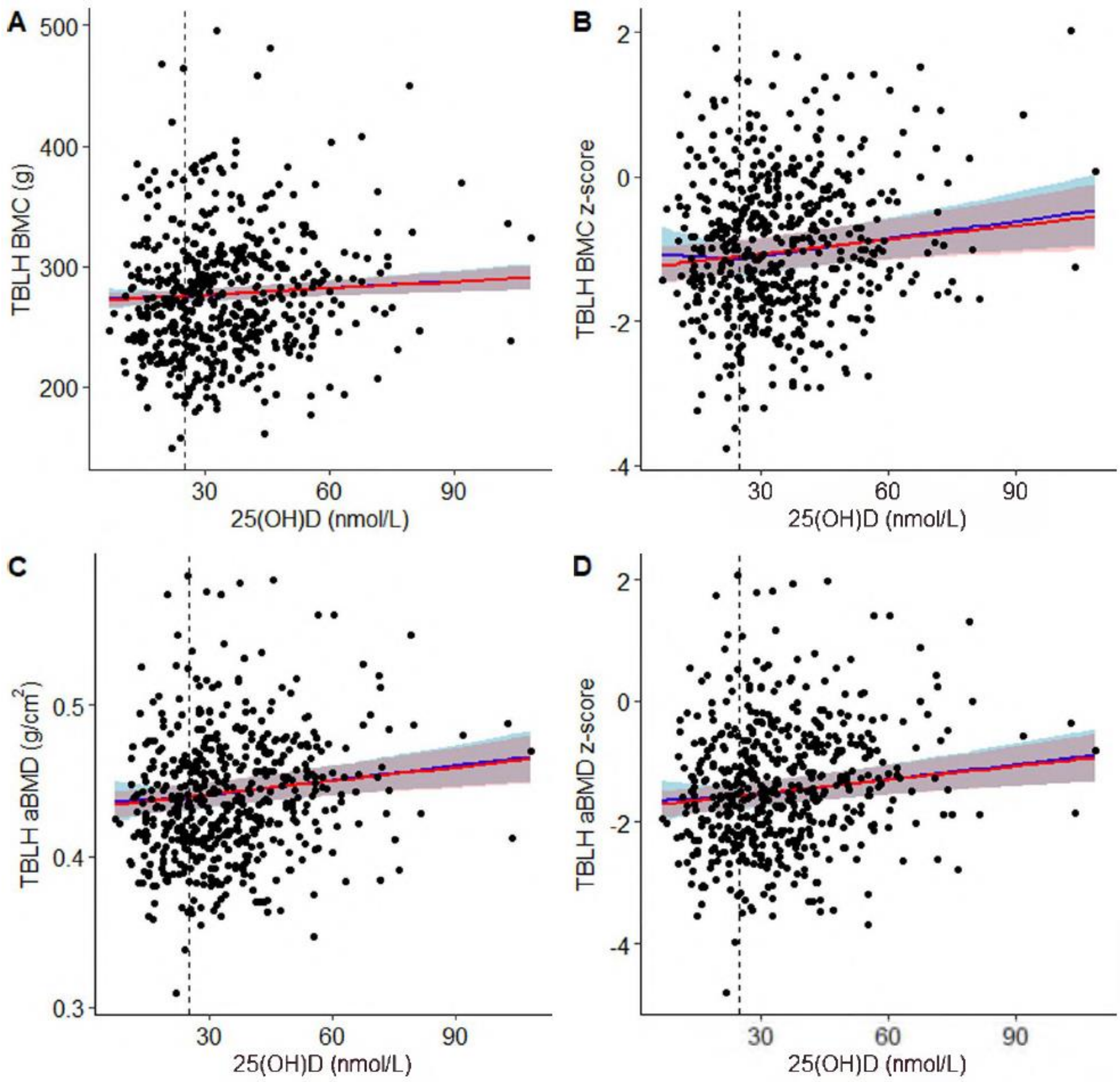

Supplementary Figure 6: Association between 25(OH)D and TBLH BMC, BMC z-score, aBMD and aBMD z-score (*n*=524) based on multivariable-adjusted (unsegmented) linear regression (red) and spline (blue) models. **(A)** Association between 25(OH)D and BMC. (**B)** Association between 25(OH)D and BMC z-score. (**C)** Association between 25(OH)D and aBMD. **(D)** Association between 25(OH)D and aBMD z-score. Covariates included the following hypothesized confounders: child sex, BMI-for-age z-score, height-for-age z-score, whole-blood hemoglobin concentration, daily frequency of dairy items consumed, season of blood draw, maternal education attainment, household asset index and the maternal prenatal and postpartum intervention group assigned at MDIG trial enrolment. Models (A) and (B) were additionally adjusted for TBLH bone area. For the spline models, the knot was placed at 25 nmol/L of 25(OH)D. For all outcomes, the linear model was considered as best model fit based on lowest or negligible differences in AIC. 25(OH)D, 25-hydroxyvitamin D; aBMD,

areal bone mineral density; AIC, Akaike's Information Criteria; BMC, bone mineral content; TBLH, total-body-less-head.

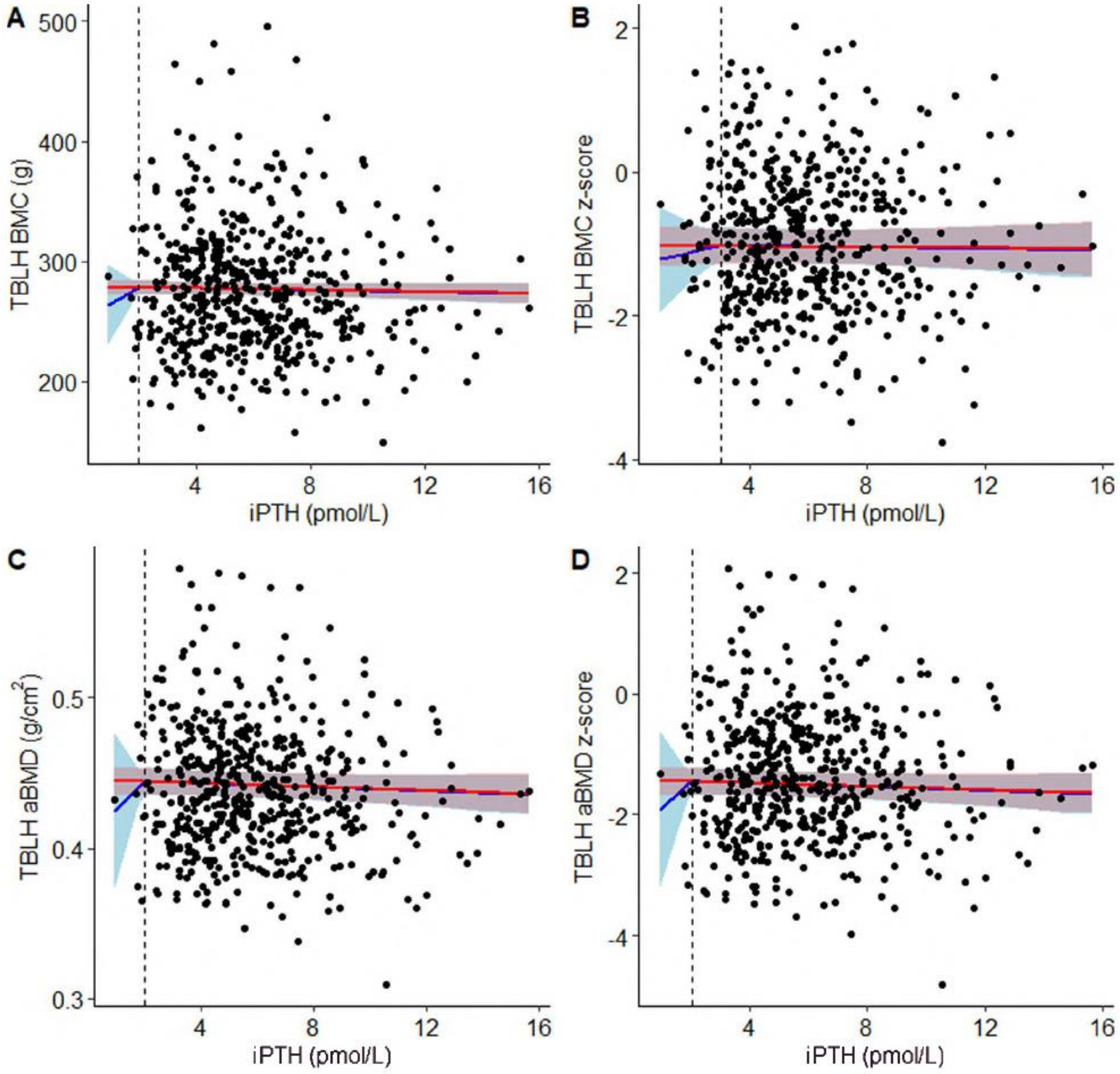

Supplementary Figure 7: Association between iPTH and TBLH BMC, BMC z-score, aBMD and aBMD z-score (*n*=524) based on multivariable-adjusted (unsegmented) linear regression (red) and spline (blue) models. (**A)** Association between iPTH and BMC. **(B)** Association between iPTH and BMC z-score. (**C)** Association between iPTH and aBMD. (**D)** Association between iPTH and aBMD z-score. Covariates included the following hypothesized confounders: child sex, BMI-for-age z-score, height-for-age z-score, serum 25-hydroxyvitamin D concentration, whole-blood hemoglobin concentration, daily frequency of dairy items consumed, season of blood draw, maternal education attainment, household asset index and the maternal prenatal and postpartum intervention group assigned at MDIG trial enrolment. Models (A) and (B) were additionally adjusted for TBLH bone area. For each of the spline models, the dashed line represents the knot placement. In all models, the (unsegmented) linear model was considered as best model fit based on lowest AIC. aBMD, areal bone

mineral density; AIC, Akaike's Information Criteria; BMC, bone mineral content; iPTH, intact parathyroid hormone; TBLH, total-body-less-head.

Supplementary Table 1: Associations of 25(OH)D with iPTH and DXA-derived bone outcomes in unadjusted regression models[1].

	Full cohort (*n*=534)			
	**β**	**95% CI**	***P***	**AIC**
**iPTH (pmol/L)**				
**Linear regression**	-0.04	-0.06 to -0.03	<0.001	2451
**Spline model**				
25(OH)D < 25 nmol/L	-0.17	-0.23 to -0.10	<0.001	2438
25(OH)D ≥ 25 nmol/L	-0.02	-0.04 to -0.01	0.003	
**TBLH BMC (g)**				
**Linear regression**	0.39	0.10 to 0.68	0.008	5735
**Spline model**				
25(OH)D < 25 nmol/L	0.06	-1.3 to 1.4	0.9	5737
25(OH)D ≥ 25 nmol/L	0.45	0.09 to 0.80	0.015	
**TBLH BMC z-score**				
**Linear regression**	0.008	0.003 to 0.014	0.002	1487
**Spline model**				
25(OH)D < 25 nmol/L	-0.011	-0.036 to 0.014	0.4	1487
25(OH)D ≥ 25 nmol/L	0.012	0.005 to 0.018	0.001	
**TBLH aBMD (g/cm^2)**				
**Linear regression**	0.0004	0.0002 to 0.0007	0.0002	-1886
**Spline model**				
25(OH)D < 25 nmol/L	0.00043	-0.0006 to 0.0015	0.4	-1884
25(OH)D ≥ 25 nmol/L	0.00044	0.0002 to 0.0007	0.003	
**TBLH aBMD z-score**				
**Linear regression**	0.01	0.005 to 0.016	<0.001	1552
**Spline model**				
25(OH)D < 25 nmol/L	0.014	-0.013 to 0.040	0.3	1554
25(OH)D ≥ 25 nmol/L	0.001	0.003 to 0.017	0.007	

[1] Estimates represent the change in the bone outcome variable per 1 nmol/L increase in 25(OH)D. Lower AIC indicated better model fit. If no differences in AIC between the (unsegmented) linear and spline model, the linear model was chosen. 25(OH)D, 25-hydroxyvitamin D; aBMD, areal bone mineral density; AIC, Akaike's Information Criteria; BMC, bone mineral content; DXA, dual-energy x-ray absorptiometry; iPTH, intact parathyroid hormone; TBLH, total-body-less head.

Supplementary Table 2: Associations between iPTH and DXA-derived bone outcomes in unadjusted regression models[1].

	Full cohort (*n* = 534)		
	**β**	**95% CI**	***P***
**TBLH BMC (g)**	-1.1	-2.9 to 0.7	0.2
**TBLH BMC z-score**	-0.014	-0.048 to 0.019	0.4
**TBLH aBMD (g/cm^2)**	-0.002	-0.003 to -0.0002	0.025
**TBLH aBMD z-score**	-0.03	-0.065 to 0.006	0.1

[1]Estimates represent the change in the bone outcome variable per 1 pmol/L increase in iPTH. aBMD, areal bone mineral density; BMC, bone mineral content; DXA, dual-energy x-ray absorptiometry; iPTH, intact parathyroid hormone; TBLH, total-body-less head.
